# Superconductivity up to 243 K in the yttrium-hydrogen system under high pressure

**DOI:** 10.1038/s41467-021-25372-2

**Published:** 2021-08-20

**Authors:** Panpan Kong, Vasily S. Minkov, Mikhail A. Kuzovnikov, Alexander P. Drozdov, Stanislav P. Besedin, Shirin Mozaffari, Luis Balicas, Fedor Fedorovich Balakirev, Vitali B. Prakapenka, Stella Chariton, Dmitry A. Knyazev, Eran Greenberg, Mikhail I. Eremets

**Affiliations:** 1 Max-Planck-Institut für Chemie, Mainz, Germany; 2 Institute of Solid State Physics Russian Academy of Sciences, Chernogolovka, Moscow District Russia; 3 National High Magnetic Field Laboratory, Florida State University, Tallahassee, FL USA; 4 NHMFL, Los Alamos National Laboratory, MS E536, Los Alamos, NM USA; 5 Center for Advanced Radiation Sources, University of Chicago, Chicago, IL USA; 6 Max-Planck-Institut für Mikrostrukturphysik, Halle (Saale), Germany

**Keywords:** Superconducting properties and materials, Electronic properties and materials

## Abstract

The discovery of superconducting H_3_S with a critical temperature *T*_*c*_∼200 K opened a door to room temperature superconductivity and stimulated further extensive studies of hydrogen-rich compounds stabilized by high pressure. Here, we report a comprehensive study of the yttrium-hydrogen system with the highest predicted *T*_*c*_s among binary compounds and discuss the contradictions between different theoretical calculations and experimental data. We synthesized yttrium hydrides with the compositions of YH_3_, YH_4_, YH_6_ and YH_9_ in a diamond anvil cell and studied their crystal structures, electrical and magnetic transport properties, and isotopic effects. We found superconductivity in the *Im-3m* YH_6_ and *P6*_3_*/mmc* YH_9_ phases with maximal *T*_*c*_s of ∼220 K at 183 GPa and ∼243 K at 201 GPa, respectively. *Fm-3m* YH_10_ with the highest predicted *T*_*c* _> 300 K was not observed in our experiments, and instead, YH_9_ was found to be the hydrogen-richest yttrium hydride in the studied pressure and temperature range up to record 410 GPa and 2250 K.

## Abstract (web summary)

The discovery of high temperature superconductivity in hydrogen-rich compounds stimulates further extensive studies. Here, the authors report superconductivity in pressurized yttrium-hydrogen system with highest predicted *T*_c_ among binary compounds.

## Introduction

High-temperature superconductivity (HTSC) is of great interest and importance because superconductors operating under ambient or technologically accessible pressure and temperature conditions can vastly improve many areas of technology. The conventional theories of Bardeen–Cooper–Schrieffer^1^ and Migdal–Eliashberg^2,3^ indicate that superconductivity, even at room temperature, cannot be excluded. However, for a long time, the critical temperature of conventional superconductors has been limited to *T*_c_ = 39 K in MgB_2_^4^. The discovery of unconventional superconductivity in the cuprates family has provided a strong motivation to further search for superconducting materials at even higher temperatures. Despite great efforts, superconductivity soon reached its limit in *T*_c_ at ~133 K^5^ (~164 K under pressure^6^). A microscopic theory of high-*T*_c_ unconventional superconductors is still lacking, which hampers further advances in this field.

The breakthrough and tremendous progress for reaching HTSC came with the discovery of the “Earth temperature superconductivity” at 203 K (−70 °C) in H_3_S at ∼150 GPa^7^. This achievement was based on the general assumption by Ashcroft, who suggested that hydrogen-dominant materials are promising candidates for HTSC^8^, which has then been greatly supported by modern ab initio computational methods for predicting the crystal structure and properties of novel materials^9–11^. This work showed a clear route towards room-temperature superconductivity and initiated extensive high-pressure studies of hydrides, which are conventional phonon-mediated superconductors. Soon *T*_c_ reached 252 K in LaH_10_ at 170 GPa^12^ (*T*_c_ = 260 K was claimed in Somayazulu et al.^13^), and very recently, *T*_c_ = 287 K was reported for the ternary carbon–sulfur–hydrogen system^14^. In addition, many other superconductors with high *T*_c_s were found (see Review^15^ and the many refs. within).

While the claim of room-temperature superconductivity^14^ is waiting for its verification and clarification of the composition and crystal structure of the phase responsible for such high *T*_*c*_, the superconductivity in H_3_S and LaH_10_ compounds has been independently reproduced by several groups. For instance, the superconducting transitions in H_3_S were confirmed with reported *T*_c_s between ∼183 and 200 K^16–19^, and superconductivity in LaH_10_ was verified in close agreement at ∼250 K^13,20,21^.

Binary hydrides remain the most promising system for fruitful synergy between theory and experiment. Nevertheless, there is a discrepancy between the measured and predicted *T*_c_s and phase diagrams, and new experimental data are crucial for further improvement of the computational methods. Recent theoretical reports provide convincing predictions for HTSC in the yttrium-hydrogen system. According to the calculations, *T*_*c*_ should be as high as 303 K at 400 GPa^22^ or 305−326 K at 250 GPa^23^ in face-centred cubic (*fcc)* YH_10_. In addition to YH_10_, there are other phases with high calculated *T*_c_s, which are predicted to be stable at lower pressures, e.g., hexagonal close packed (*hcp*) YH_9_ with a *T*_*c*_ of 253−276 K stable at 200 GPa^22^ and body-centred cubic (*bcc*) YH_6_ with a *T*_c_ of 251−264 K stable at 110 GPa^24^.

In the present work, we report an experimental study of the yttrium-hydrogen system at high pressures and two superconducting *bcc*-YH_6_ and *hcp*-YH_9_ phases. Both phases have the same crystal structures as those predicted by the calculations^22–24^; however, the observed *T*_c_s are significantly lower than the calculated values by ~30 K. We could not synthesise *fcc*-YH_10_, which has the highest computed *T*_c_ among the predicted yttrium hydrides. Instead, we revealed that *hcp*-YH_9_ is the persistent hydrogen-richest yttrium hydride in the wide pressure-temperature domain up to 410 GPa and 2250 K – under the same conditions where the thermodynamic stability of the *fcc*-YH_10_ phase was predicted^22^. Here, we also discuss the contradictions between different experimental data for the yttrium-hydrogen system. Our preliminary report^25^ and the present more comprehensive data are in good agreement with the data from Troyan et al.^26^ but evidently contradict the claim of *T*_c_ = 262 K at 182 GPa reported by Snider et al.^27^.

## Results and discussion

### Synthesis of samples

We prepared various yttrium hydrides in a diamond anvil cell (DAC) by compressing either yttrium metal in H_2_/D_2_ (9 samples), yttrium trihydride YH_3_/YD_3_ in H_2_/D_2_ (9 samples), or YH_3_ with NH_3_BH_3_ (7 samples). The details for the 31 different samples synthesised and analysed in the present study are summarised in Supplementary Table 1.

In our experiments_,_ yttrium metal reacted with the surrounding hydrogen fluid at 17 GPa at room temperature, which was the lowest pressure at which X-ray diffraction patterns were collected (samples 26 and 27; Supplementary Fig. 1). This chemical reaction occurs even at lower pressures of ∼1 GPa according to refs. ^28,29^.

**Fig. 1:**
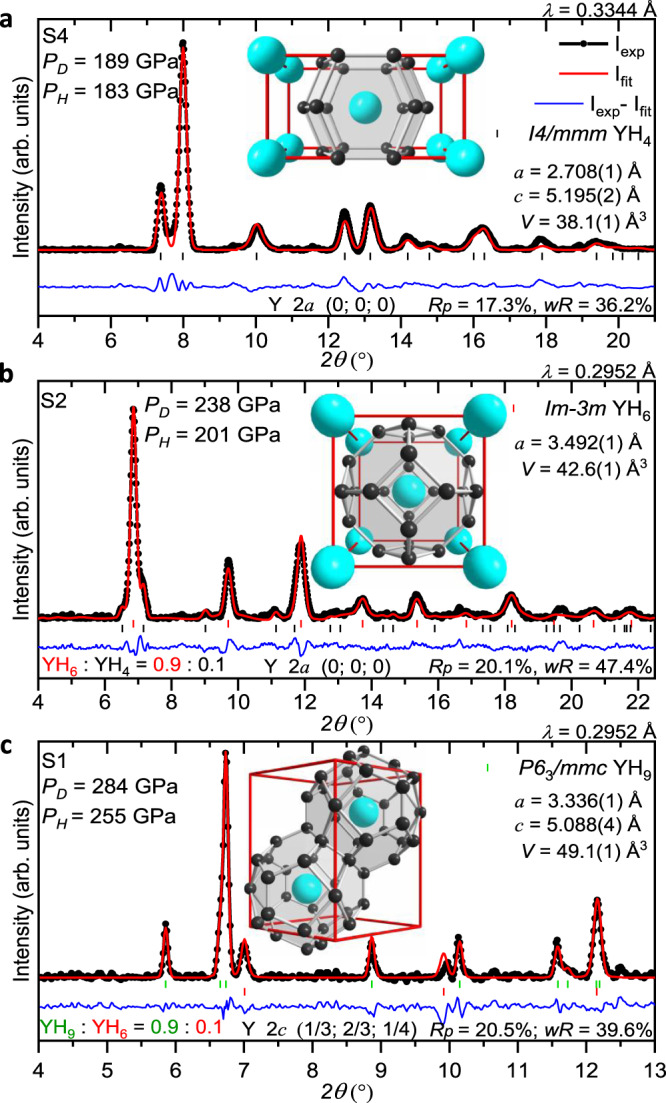
X-ray powder diffraction patterns of the synthesised yttrium hydrides. **a***I4/mmm* YH_4_ phase in sample 4 (S4) after pulsed laser heating at *P*_H_ = 183 GPa; **b** *Im-3m* YH_6_ phase in unheated sample 2 (S2) at *P*_H_ = 201 GPa with a *T*_c_ of ∼211 K; **c** *P6*_3_*/mmc* YH_9_ phase in sample 1 (S1) after pulsed laser heating at *P*_H_ = 255 GPa with a *T*_c_ = 235 K. See Supplementary Table 1 for details. *P*_H_ and *P*_D_ correspond to the pressures estimated by H_2_ (D_2_) vibron scale^54^ and diamond scale^55^, respectively. The black circles and red and blue curves correspond to the experimental data, Rietveld refinement fits and residues, respectively. The black, red and green ticks indicate the calculated peak positions for the *I4/mmm* YH_4_, *Im-3m* YH_6_ and *P6*_3_*/mmc* YH_9_ phases, respectively. The weight fractions for the phases, refined lattice parameters and coordinates for Y atoms are shown for each refinement. The fragments of the crystal structure with the characteristic YH_18_, YH_24_ and YH_29_ coordination polyhedra (cages) are shown as insets. The large cyan and small black spheres show the positions of the Y and H atoms in the crystallographic unit cells according to Peng et al.^22^.

**Fig. 2:**
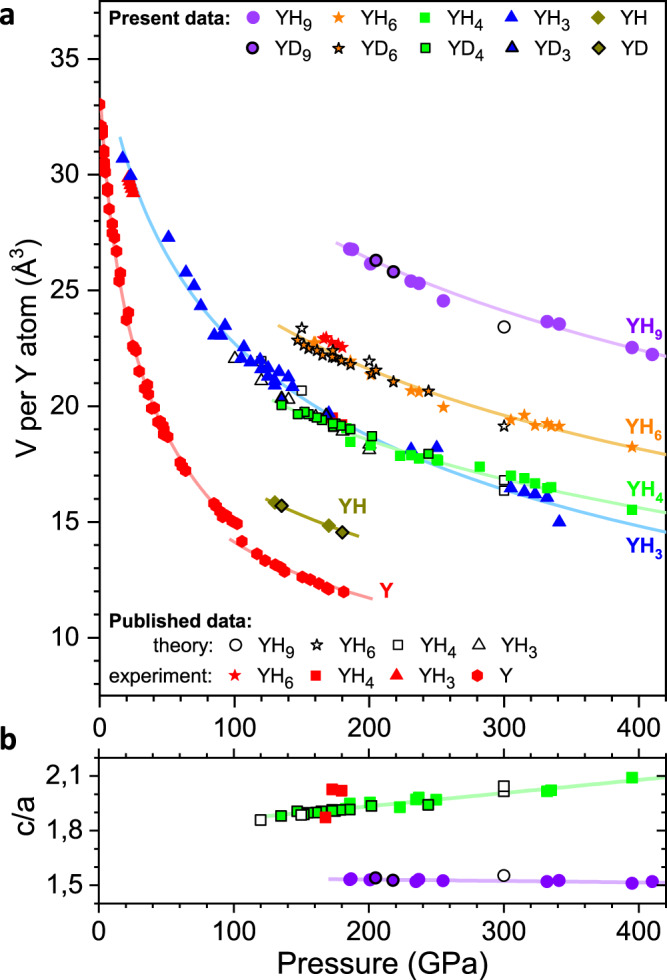
Volume of various synthesised yttrium hydrides as a function of pressure. **a** The experimental data for the *P6*_3_*/mmc* YH_9_, *Im-3m* YH_6_, *I4/mmm* YH_4_, *Fm-3m* YH_3_ and *Fm-3m* YH phases are shown as filled violet circles, orange stars, green squares, blue triangles and dark yellow rhombuses, respectively. The data for the corresponding yttrium deuterides are outlined in black. Open black circles, stars, squares and triangles correspond to the theoretically predicted structures for YH_9_^22^, YH_6_^22–24,26^, YH_4_^22–24,26^ and YH_3_^31,32^, respectively. Experimental data for pure Y^33,63^, YH_3_^59^, YH_4_^26^ and YH_6_^26^ taken from the literature are depicted by the red symbols. Solid curves correspond to the Vinet^30^ equation of state fitting. The pressure was estimated from the frequency of the H_2_ (D_2_) vibron^54^ for the samples with excess H_2_ (D_2_) and from the high-frequency edge of the Raman line from the stressed diamond anvil^55^ for the remainder of our samples. **b** The pressure dependence for the lattice parameter ratio *c*/*a* in the *P6*_3_*/mmc* and *I4/mmm* phases with linear fits.

Yttrium hydrides with a higher hydrogen content require much higher pressures for their formation. We synthesised body-centred tetragonal (*bct*) YH_4_/YD_4_ and *bcc*-YH_6_/YD_6_ within a pressure range of 160–175 GPa (Figs. 1 and 2) after heating YH_3_/YD_3_ in H_2_/D_2_ at ∼1500 K with the aid of a pulsed laser (samples 5, 7 and 24). Both phases can also be formed by exposing the reactants to higher pressures of ~200–244 GPa at room temperature for several weeks (samples 1, 2, 6, 9 and 10).

Both *bct-*YH_4_/YD_4_ and *bcc-*YH_6_/YD_6_ were observed within a wide pressure range of ∼160–395 GPa (Fig. 2). Laser heating of these phases with excess H_2_/D_2_ at pressures above 185 GPa results in the formation of the *hcp*-YH_9_/YD_9_ phase (samples 1, 2, 3, 6, 7, 10, 17–19 and 21–23).

### X-ray diffraction studies

The crystal structures of novel yttrium hydrides were determined by X-ray powder diffraction. The Rietveld refinements for the typical powder diffraction patterns of the *I4/mmm* YH_4_, *Im-3m* YH_6_ and *P6*_3_*/mmc* YH_9_ phases are shown in Fig. 1. The lattice parameters of these phases were determined at 135–410 GPa for a series of different samples and are listed in Supplementary Tables 1–5. The volumes of the crystal lattices of the synthesised yttrium hydrides are summarised in Fig. 2a as a function of pressure and are well approximated by the Vinet^30^ equation of state (fitting parameters are given in Supplementary Table 6). The lattice volumes of *bct-*YH_4_, *bcc*-YH_6_ and *hcp*-YH_9_ (Fig. 2a) and the *c*/*a* ratios of *bct-*YH_4_ and *hcp*-YH_9_ (Fig. 2b) agree well with the theoretical predictions^22–24,26,31,32^, which justifies the assignment of compositions for the hydrides with H/Y > 3.

We also independently estimated the compositions of new yttrium hydrides from the X-ray diffraction data by analysing the hydrogen-induced volume expansion V_H_. In contrast to the lanthanum-hydrogen system^12^, in which the *V*_H_ of ∼1.8 Å^3^/H(D) atom is nearly identical in all lanthanum hydrides at approximately 150 GPa, the corresponding volume expansion is anomalously large in YH_3_ and differs from that in the yttrium hydrides with higher hydrogen content. As a result, *bct*-YH_4_/YD_4_ and *fcc*-YH_3_/YD_3_ have almost the same lattice volumes within a pressure range of ∼150–250 GPa. Using the extrapolated equation of state for pure yttrium^33^, the value of *V*_H_ in *bct*-YH_4_(YD_4_) is ∼1.7–1.8 Å^3^/H(D) atom at 180–200 GPa and is comparable with the values measured in other hydrogen-rich metal hydrides^12,34–37^. The derived values provide the estimation for compositions of the new *bcc* and *hcp* phases as YH_5.7(3)_ and YH_8.5(5)_.

The synthesis of YH_10_, which, according to theory, is the most promising candidate for the highest *T*_c_ of 305−326 K^23^, was of particular interest for the present study. The calculations on the stability of *fcc-*YH_10_ provide contradictory results. Some predictions show that this phase becomes stable at 250 GPa^23^, which is an apparent contradiction with our observations. Other calculations suggest that at *T* = 0 K, YH_10_ is thermodynamically unstable at any pressure^22^. However, the difference in the Gibbs free energies between *fcc*-YH_10_ and *hcp-*YH_9_ should decrease at higher temperatures, and *fcc*-YH_10_ should become more favourable at temperatures above 1500 K at 375 GPa or above 1100 K at 400 GPa^22^.

**Fig. 3:**
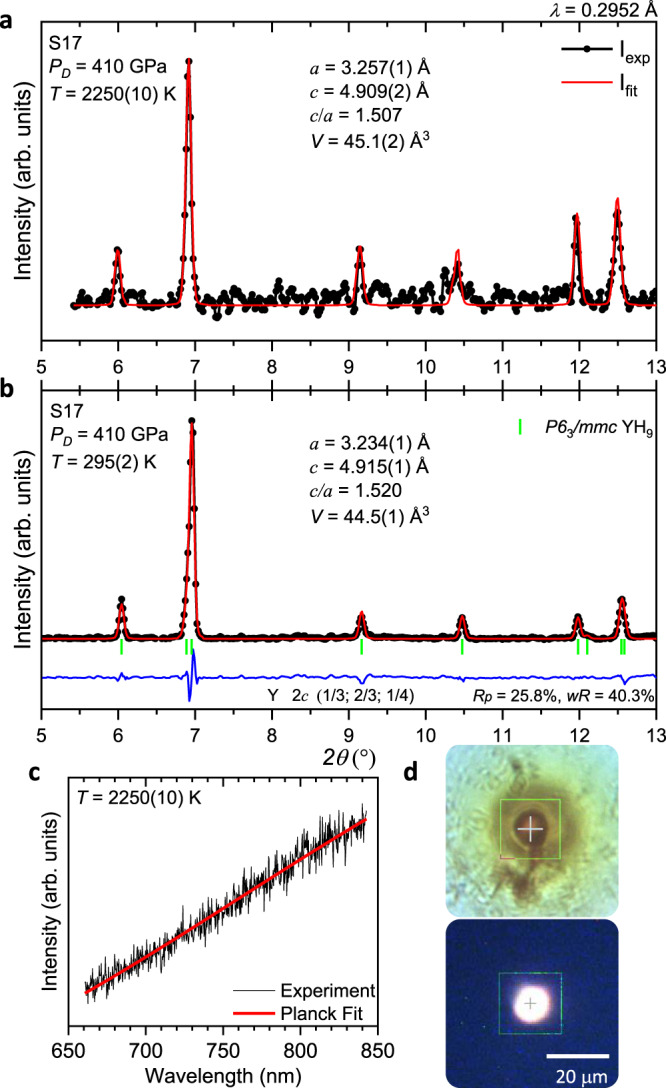
X-ray diffraction study of the yttrium-hydrogen system at extreme pressure and temperature conditions. **a** X-ray powder diffraction pattern collected from sample 17 (S17) (YH_3_ + NH_3_BH_3_) at 410 GPa directly at the moment of pulsed laser heating at 2250(10) K (black circles) and Le Bail refinement (red curve). **b** X-ray powder diffraction pattern of sample 17 at 410 GPa after subsequent quenching to ambient temperature, corresponding to the pure *P6*_3_*/mmc* YH_9_ phase. Black circles, red and blue curves correspond to the experimental data, Rietveld refinement fits and residues, respectively. Green ticks indicate the calculated peak positions for the *P6*_3_*/mmc* YH_9_ phase. **c** The thermal radiation spectrum measured during pulsed laser heating (black curve) and the fit to Planck’s radiation law (red curve). **d** Photos of the sample at ambient temperature (top) and during pulsed laser heating (bottom).

Guided by these calculations, we prepared samples of YH_3_ with NH_3_BH_3_ to study the yttrium-hydrogen system at very high pressures of ∼325–410 GPa (see Supplementary Table 1). We did not observe *fcc*-YH_10_ in the final quenched products after pulsed laser heating of the samples up to 1600–2250 K. There was no hint of the *fcc-*YH_10_ phase immediately during pulsed laser heating; only the temperature-induced thermal expansion of the crystal structure of the *hcp-*YH_9_ phase at 410 GPa and 2250(10) K was detected (Fig. 3). Notably, excess H_2_ is hard to control in experiments with NH_3_BH_3_ as the source of hydrogen. Nonetheless, it is evident that excess H_2_ was realised in sample 17 because the initial YH_3_ completely transformed into single-phase *P6*_3_*/mmc* YH_9_ after laser heating (Fig. 3). It is possible that the predicted YH_10_ exists at even higher pressures and temperatures, but an experiment at such extreme conditions is currently challenging.

### Superconductivity

**Fig. 4:**
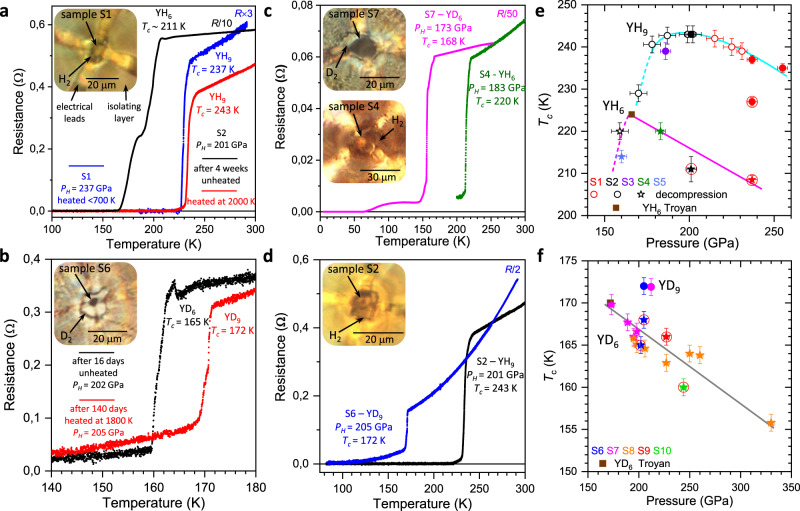
Superconducting transitions in the synthesised yttrium hydrides and deuterides. **a**, **b** The temperature dependencies of the resistance of the yttrium-hydrogen and yttrium-deuterium samples measured with a four-probe technique in a van der Pauw geometry, demonstrating the shift in *T*_c_ to higher temperatures after pulsed laser heating of the samples. In **a**, Black and red curves correspond to the temperature dependence of the resistance of the *Im-3m* YH_6_ phase, which was formed after exposing YH_3_ to H_2_ at *P*_H_ = 201 GPa for 3 weeks, and the *P6*_3_*/mmc* YH_9_ phase, which was synthesised after subsequent heating at 2000(10) K, in sample 2. The blue curve corresponds to the *P6*_3_*/mmc* YH_9_ phase synthesised at *P*_H_ = 237 GPa in sample 1 (S1). In **b**, similarly, *T*_c_ increases from 165 K for *Im-3m* YD_6_ to 172 K for *P6*_3_*/mmc* YD_9_ in sample 6 (S6) at *P*_H_ ∼ 205 GPa. **c**, **d** The temperature dependencies of the resistance of *Im-3m* YH_6_ (YD_6_) and *P6*_3_*/mmc* YH_9_ (YD_9_) synthesised in samples 2, 4, 6 and 7 (S2, S4, S6, S7) demonstrate the shift in the superconducting transitions with isotopic substitution. The absolute resistance values for some samples were multiplied by the specified constant factors for better presentation. The insets show photos of the samples and arrangements for electric transport measurements. **e**, **f** The pressure dependence of *T*_c_ for the superconducting transitions in *Im-3m* YH_6_ (stars) and *P6*_3_*/mmc* YH_9_ (circles) phases and the corresponding deuterides, respectively. Different colours represent different samples. Open symbols are the data obtained on subsequent decompression. Symbols marked by red circles are the data for unheated samples. Cyan, magenta and grey curves are the guides for the eye. Brown squares depict the data from Troyan et al.^26^. Error bars are defined the same as in Drozdov et al.^12^. The horizontal and vertical error bars correspond to the uncertainty in the precise value of the pressure (inherent error bars of the method used) and in the determination of the correct value of *T*_c_ (criteria-dependent), respectively.

The electrical resistance measurements of new yttrium hydrides revealed superconductivity with high *T*_c_s (Fig. 4). The observed superconducting transitions were unambiguously assigned to either *hcp-*YH_9_/YD_9_ or *bcc-*YH_6_/YD_6_ by analysing the phase content in several of the prepared samples (see details in Supplementary Table 1). According to the X-ray diffraction data, some samples contained variable amounts of lower hydrides, namely, *fcc*-YH_3_ and *bct-*YH_4_, originating from the areas near the electrical leads. These areas were relatively poorly heated on purpose by the pulsed laser to prevent the failure of the electrical leads on the samples. The presence of these impurities does not alter the observed HTSC in our samples (see below).

The *T*_c_ of *hcp-*YH_9_ is higher than that of the *bcc*-YH_6_ phase, as follows from the electrical measurements for sample 2 (the black and red curves in Fig. 4a). Prior to laser heating, the sample contained *bcc-*YH_6_ and exhibited a superconducting transition with a *T*_c_ of ∼211 K at 201 GPa. After heating at 2000(10) K, most of the *bcc*-YH_6_ phase transformed into *hcp*-YH_9_, and the superconducting transition shifted to a higher temperature of ∼243 K (the X-ray diffraction patterns of sample 2 are shown in Supplementary Fig. 2d, e). Identical behaviour was observed for the deuterides in sample 6 at 202 GPa (the black and red curves in Fig. 4b); i.e., the initial *bcc-*YD_6_ phase had a *T*_c_ of 165 K, and *hcp-*YD_9_, which formed after laser heating, exhibited a higher *T*_c_ of 172 K (the X-ray diffraction patterns of sample 6 are presented in Supplementary Fig. 3).

The pressure dependencies of *T*_c_ for YH_9_ and YH_6_ have a “dome-like” shape with the highest measured *T*_c_ of 243 K at 201 GPa and 224 K at 166 GPa, respectively (Fig. 4e). Similar maxima at the *T*_c_(P) dependence were previously observed in H_3_S^7^ and LaH_10_^12^ at ∼150 GPa. The decrease in *T*_c_ with increasing pressure in the *bcc-*YH_6_ and *hcp-*YH_9_ phases is likely due to either the pressure-induced stiffening of the phonon frequencies similar to that in *bcc*-H_3_S^38,39^ or the presence of a flat region on the Fermi surface and the appearance of a two-gap structure similar to that in *fcc*-LaH_10_^40,41^. The data for *bcc-*YH_6_ and YD_6_ independently measured by Troyan et al.^26^ agree with this trend. The sharp drop in *T*_c_ for YH_9_ and YH_6_ at pressures below ∼195 and ∼165 GPa, respectively, should be associated with the structural distortions and phase transformations between the high-pressure high-symmetry and low-pressure low-symmetry phases, as was recently demonstrated for H_3_S^18,42^ and LaH_10_^21,43^.

It should be noted that some samples synthesised by keeping reactants at room temperature for several weeks without laser heating (samples 1, 2, 6 and 10) exhibit *T*_c_s that are lower by ∼5–10 K in comparison to the samples prepared via laser heating-assisted synthesis (the corresponding symbols are outlined by the red circles in Fig. 4e, f). This effect should be attributed to the poorer crystallinity and homogeneity of the superconducting phase in the non-annealed samples, which manifested in the broadening of the Bragg reflections in the X-ray diffraction powder patterns (Supplementary Figs. 2 and 3). Similar behaviour was previously shown in H_3_S^7,18^.

### Isotope effect

The substitution of hydrogen by deuterium in the samples resulted in a pronounced shift in *T*_c_ to lower temperatures. The transition temperature shifted to ∼168 K for YD_6_ in sample 7 at 173 GPa and ∼172 K for YD_9_ in sample 6 at 205 GPa (Fig. 4c, d). This isotope effect supports the conventional phonon-assisted mechanism of superconductivity. Using the *T*_c_ values measured for *hcp*-YH_9_/YD_9_ and *bcc*-YH_6_/YD_6_ within the pressure range of 183–205 GPa (samples 2, 4, 6 and 7), we calculated the isotope effect coefficient, $\alpha =-\frac{{{dlnT}}_{{{{\mathrm{c}}}}}}{{dlnM}}$, where *M* is the atomic mass, to be 0.39 for the *bcc*-YH_6_/YD_6_ phase and 0.50 for the *hcp*-YH_9_/YD_9_ phase. The isotope coefficient value for *bcc*-YH_6_/YD_6_ is smaller than the maximal expected *BCS* value of 0.5 for a harmonic case. This result likely stems from the anharmonic effects and the contribution of acoustic phonons to electron-phonon coupling.

### Resistance measurements under high magnetic fields

**Fig. 5:**
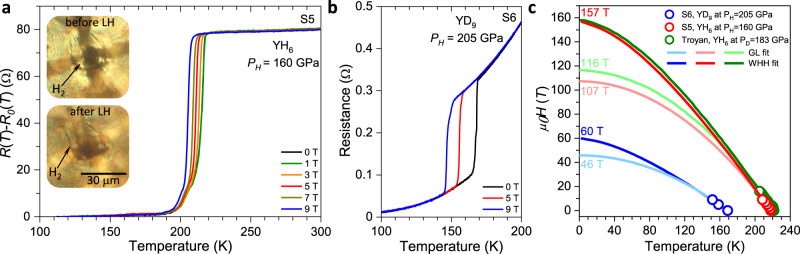
Temperature dependence of the resistance for the *Im-3m* YH_6_ and *P6*_3_*/mmc* YD_9_ phases under external magnetic field. **a**, **b** DC field measurements for the *Im-3m* YH_6_ phase at *P*_H_ = 160 GPa in sample 5 (S5) and the *P6*_3_*/mmc* YD_9_ phase at *P*_H_ = 205 GPa in sample 6 (S6), respectively. **c** Fits of the superconducting upper critical field *H*_c2_ to the Werthamer–Helfand–Hohenberg (WHH) and Ginzburg–Landau (GL) formalisms. Red and blue circles denote the *H*_c2_s measured for *Im-3m* YH_6_ at *P*_H_ = 160 GPa and *P6*_3_*/mmc* YD_9_ phase at *P*_H_ = 205 GPa, respectively. The dark and light curves are the WHH and GL fits to the experimental data. Green circles and green curves are the data for *Im-3m* YH_6_ phase at *P*_D_ = 183 GPa from Troyan et al.^26^.

In addition to the observed drops in the resistance to a zero value and the isotope effect, the onset of superconducting order was independently verified by the magneto-transport measurements under magnetic fields up to 9T. While the magnetic field has a negligible effect on the resistance of the normal metal, *T*_c_ is strongly reduced as the magnetic field increases, and superconductivity is completely suppressed at fields above the upper critical field *H*_c2_. *H*_c2_ is the most direct probe of the coherence length of the superconducting order parameter $\xi =\sqrt{\frac{{{{{\rm{\phi }}}}}_{0}}{2{{{\rm{\pi }}}}{{{{\rm{H}}}}}_{c2}}}$, where ϕ_0_ is the magnetic flux quantum. Figure 5a, b show the dependence of the superconducting transition in samples 5 and 6 as a function of external magnetic field. To estimate the *H*_c2_ and *ξ* at zero temperature, we plotted the dependence of *T*_c_ on the applied external magnetic field, following the criterion of 90% of the resistance in the metallic state (Fig. 5c). The temperature dependence of *H*_c2_ can be approximated by the Ginzburg–Landau (GL) equation^44^ for $\frac{{T}_{{{{\mathrm{c}}}}}-T}{{T}_{{{{\mathrm{c}}}}}}\ll 1$ and more accurately by the Werthamer–Helfand–Hohenberg (WHH)^45^ model for all temperatures. The light and dark curves in Fig. 5c show the results of the fits for the experimental values of *H*_c2_(*T*) to the GL and WHH relations. These fits yield *H*_c2_^WHH^(0 K) = 60 T (*H*_c2_^GL^(0 K) = 46 T) for *hcp*-YD_9_ and *H*_c2_^WHH^(0 K) = 157 T (*H*_c2_^GL^(0 K) = 107 T) for *bcc*-YH_6_. The latter values are in good agreement with the estimate of *H*_c2_(0 K) = 116–158 T by Troyan et al.^26^. The corresponding coherence length *ξ* (0 K) in *bcc*-YH_6_ and *hcp*-YD_9_ are 1.45–1.75 nm and 2.3–2.7 nm, respectively.

We estimated *H*_c2_^WHH^ (0 K) = 120 T (*H*_c2_^GL^ (0 K) = 92 T) for *hcp*-YH_9_ using the relation of ${H}_{{{{\mathrm{c}}}}2} \sim \,{(\frac{{T}_{{{{\mathrm{c}}}}}}{{v}_{{{F}}}})}^{2}$ and assuming the same Fermi velocities *υ*_F_ in YH_9_ and YD_9_ counterparts. Generally, higher *T*_c_ values correlate with higher *H*_c2_(0 K) values in the studied hydride superconductors, e.g. *H*_c2_ = 144 T and *T*_c_ = 250 K were observed in LaH_10_^12,21^, *H*_c2_ = 88 T and *T*_c_ = 197 K in H_3_S^16^, *H*_c2_ = 45 T and *T*_c_ = 153 K in ThH_10_ and *H*_c2_ = 38 T and *T*_c_ = 145 K in ThH_9_^36^, *H*_c2_ = 29 T and *T*_c_ = 98 K in CeH_10_^46^, and *H*_c2_ = 11 T and *T*_c_ = 68 K in SnH_x_^47^. Conversely, the *hcp*-YH_9_ phase has higher *T*_c_ = 243 K but lower *H*_c2_ comparing with *bcc*-YH_6_ phase. This is likely caused by the difference in electronic band structure in these phases, and further theoretical calculations are required to explain this anomaly.

We found that despite a substantial difference in the *H*_c2_(0 K) values for YD_9_ and YH_6_ samples, the Fermi velocities *υ*_F_ estimated via the BCS relation: $\xi =0.18\frac{\hslash {\upsilon }_{{{{\rm{F}}}}}}{{k}_{{{{\mathrm{B}}}}}{T}_{{{{\mathrm{c}}}}}}$ are quite similar, i.e. 2.85 × 10^5^ and 2.3 × 10^5^ m/s, respectively. Similar *υ*_F_ values were reported for other superconductors of so-called “superhydride” family including SnH_x_^47^ and LaH_10_^21^. This indicates that the dispersion of the charge carriers contributing to the superconductivity does not significantly change between different superconducting hydrides. Interestingly, nearly constant values of *υ*_F_ were also revealed for various unconventional high-temperature superconductors of the cuprates family^48^.

### Stability range of yttrium hydrides

The predicted crystal structures of the YH_3_, YH_4_, YH_6_ and YH_9_ compositions are in excellent agreement with the experimental results. To assess the accuracy of the calculations, we compared the predicted formation pressures for these novel hydrides with our experimental observations. The problem of determining the equilibrium pressure in experiments is often exacerbated by the presence of large baric hysteresis between the formation and decomposition pressures. It is generally accepted that the equilibrium pressure in most metal-hydrogen systems is much closer to the decomposition pressure of the high-pressure phase^49,50^. Decreasing pressure in sample 2 resulted in the decomposition of YH_9_ into *bcc*-YH_6_ at 159 GPa (Supplementary Fig. 2f), which is considerably higher than the predicted YH_9_ equilibrium formation pressure of 100 GPa^22^. The sharp drop in *T*_c_ for *hcp*-YH_9_ at ∼185 GPa (open black star in Fig. 4e) indicates that this phase is dynamically unstable below this pressure. Decreasing pressure in sample 24 resulted in a decomposition of *bcc*-YD_6_ at ∼135 GPa, whereas *bct*-YD_4_ was stable down to at least ∼135 GPa (Supplementary Tables 1 and 3). This result is in reasonable agreement with the predicted equilibrium formation pressure of 110 GPa for both phases^24^.

The predicted *T*_c_s of 251–264 K for *bcc*-YH_6_^24^ and 253–276 K^22^ for the *hcp*-YH_9_ phase are ~30 K higher than the present experimental values. Recent calculations^26^ for the *bcc*-YH_6_ phase, which accounted for the anharmonicity, demonstrate a *T*_c_ of 236–247 K, which is still significantly higher than the experimental value. In addition, we did not observe superconductivity in *fcc*-YH_3_ upon cooling down to 5 K in the pressure range of 15–180 GPa or in *bct-*YH_4_ upon cooling down to 78 K at 250 GPa (Supplementary Fig. 4), while both phases were predicted to be superconductors with *T*_c_s of 40 K^51^ and 84–95 K^24,32^, respectively.

### Comparison with other works

In the recent report, Snider et al^27^. claimed a significantly higher maximum *T*_c_ = 262 K at 182(8) GPa in the yttrium-hydrogen system. Their values of *T*_c_s measured in the pressure range of ∼134–187 GPa and the pressure dependence of *T*_c_ contradict the results of both the present work and Troyan et al.^26^ (Supplementary Fig. 5). This strong disagreement raises a question about the material studied in Snider et al.^27^. Unfortunately, the lack of any X-ray structural characterisation of the samples in Snider et al.^27^ makes the direct comparison impossible. The superconductivity was putatively assigned to *P6*_3_*/mmc* YH_9_ based on a comparison of the measured and computed Raman spectra^27^. However, Raman spectroscopy is not a reliable method for the identification of a crystal structure. Moreover, *bct*-YH_4_, *bcc*-YH_6_ and *hcp*-YH_9_ are good metals and could not account for the observed Raman spectra. Presently, we observed Raman spectra only for mixtures with a hydrogen-depleted *fcc* phase with a composition close to YH_1_/YD_1_, which was formed during the compression of the initial YH_2.92(5)_ and YD_2.87(5)_ above ∼100 GPa (see Methods section for details and Supplementary Fig. 6). Furthermore, the superconducting transitions measured in Snider et al.^27^. below ∼185 GPa cannot be assigned either to *hcp-*YH_9_/YD_9_ because it is unstable in this pressure range or to *bcc*-YH_6_ because this phase has a significantly lower *T*_c_.

### Summary

We report on the superconductivity in *hcp-*YH_9_ with a maximum *T*_c_ of 243 K at 201 GPa, which is the second highest *T*_c_ measured for the family of transition element superhydrides, and *bcc-*YH_6_ with a *T*_c_ of 220 K at 183 GPa. At higher pressure, both phases demonstrate a decrease in *T*_c_. The decrease in *T*_c_ under external magnetic fields additionally confirms the superconductivity in novel yttrium superhydrides, and the isotopic shift in the superconducting transition in deuterides to lower temperatures supports the conventional phonon-assisted mechanism of superconductivity. We found good agreement between the predicted and experimental crystal structures and the *V(P)* dependencies of the synthesised hydrides. However, the measured *T*_c_s for *bcc*-YH_6_ and *hcp*-YH_9_ are markedly lower than the computed values of 251−264 K for YH_6_^24^ and 253−276 K for YH_9_^22^. We did not find the *fcc*-YH_10_ phase despite extensive trials at pressures up to 410 GPa and temperatures up to 2250 K.

## Methods

### Diamond anvil cell

Typically, DACs have diamonds bevelled at 8° to a diameter of ~250 µm with a culet size of ~15−35 µm. The diamond anvils had a toroidal profile for the samples pressurised over ∼200 GPa, which was machined by a focused beam of xenon ions (FERA3, Tescan). Four tantalum or tungsten leads covered by gold were deposited onto the surface of one diamond anvil in a van der Pauw geometry. A metallic gasket (T301 stainless steel) was thoroughly isolated from the sputtered leads by a non-conducting layer prepared from a mixture of low-viscosity epoxy resin and a fine powder of either CaF_2_, CaO, MgO, CaSO_4_, cBN or Al_2_O_3_. The insulating gasket was pressed to a thickness of 3–5 μ, and a hole with a diameter of ~2/3 of the culet size was drilled by a pulsed ultraviolet laser.

### Preparation of samples

Yttrium hydrides were synthesised in situ in a DAC via a direct reaction between either metallic yttrium (99.9%, Sigma Aldrich)) and H_2_ (99.999%) (D2, 99.75%, Spectra Gases) or, alternatively, YH_3_ (YD_3_) and H_2_ (D_2_) at pressures up to ~250 GPa. As an alternative source of hydrogen, NH_3_BH_3_ (97%, Sigma Aldrich) was used at pressures of 250–410 GPa. The Y or YH_3_ (YD_3_) pieces were typically 5–15 µm in diameter and 1–2 µm thick. The samples were handled in an inert Ar or N_2_ atmosphere with residual O_2_ and H_2_O contents of <0.1 ppm to prevent oxidation. The procedure of hydrogen gas clamping and laser heating-assisted synthesis was the same as that for lanthanum hydrides^12^. One-side heating of the sample was performed with the aid of a pulsed YAG laser. Elevated temperatures accelerate the diffusion of hydrogen into the metal; however, hot hydrogen can easily break diamond anvils by penetrating deep into microcracks at the surface of diamond. We avoided this by the rigorous polishing and etching of diamonds.

All samples synthesised and studied in the present work are summarised in Supplementary Table 1.

The YH_3_ and YD_3_ samples used as the initial materials in the DAC experiments were synthesised using bulk yttrium metal that was preliminarily annealed in a vacuum of ~10^−3^ Torr at 400 °C and then exposed to H_2_(D_2_) gas at a pressure of ~100 bars at 400 °C for 4 h and then at 200 °C for 24 h in a high-pressure Sievert-type apparatus^52^. According to a weighting method, the products were YH_2.92(5)_ and YD_2.87(5)_. The samples were powdered and analysed with an Empyrean X-ray diffractometer in an inert atmosphere under ambient conditions. These materials consisted of pure single-phase *hcp*-YH~_3_ and YD~_3_ (Supplementary Fig. 7). The lattice parameters of both products were in agreement with published data^53^. For brevity, these materials are referred to as YH_3_ and YD_3_ throughout the paper.

### Electrical transport measurements

DC electrical measurements were performed on cooling and warming cycles with an electrical current of 10^−5^–10^−3^ A (Keithley 6220 and 2000). The present data were taken upon warming as it yields a more accurate temperature reading; that is, the cell was warmed up slowly (0.2 K min^−1^) in a quasi-isothermal environment without coolant flow. The temperature was measured with an accuracy of ~1 K by a Si diode thermometer (Lakeshore DT-470) attached to the DAC body. The *T*_c_ was determined from the onset of superconductivity at the point of the apparent deviation of the temperature dependence of the resistance from the normal metallic behaviour.

Alongside the standard stainless steel DACs, special types of DACs with external diameters of 20 mm and 8.8 mm made of non-magnetic materials were used for measurements under external magnetic fields using a 9T Quantum Design Physical Property Measurement System (PPMS).

### Estimation of pressure

The pressure in the DACs was estimated using the H_2_ (D_2_) vibron scale^54^ if the corresponding vibron could be observed in the Raman spectra or diamond scale^55^ based on the shift of the Raman line edge of stressed diamond and marked throughout the text as *P*_H_ and *P*_D_, respectively. Typically, the second scale provides overestimated pressure values by ~5–40 GPa, which is a result of a large pressure gradient between the soft H_2_/D_2_ medium and the surrounding harder gasket. Unless otherwise stated, the pressure values displayed in the figures were estimated using the H_2_ (D_2_) vibron scale. Additionally, the pressure in samples 22, 26 and 27 was estimated using the equation of state of MgO^56^, which served as a gasket material.

### X-ray diffraction measurements

X-ray diffraction data were collected at beamline 13-IDD at GSECARS, Advanced Photon Source, using *λ*_1_ = 0.2952 Å and *λ*_2_ = 0.3344 Å, a beam spot size of ~2.5 × 2.5 µm^2^, and a Pilatus 1 M CdTe detector. A typical exposure time varied between 10 and 300 s. To examine the formation of new yttrium hydrides at 325–410 GPa at high temperatures, we collected X-ray powder diffraction patterns in situ at high temperature. Each pattern was collected by accumulating 5 × 10^5^ shots with a duration of 1 µs, which were synchronised with laser heating pulses. The temperature was determined by the thermal emission from the sample measured with a PI-MAX3 detector. Primary processing and integration of the powder patterns were performed using Dioptas software^57^. The indexing of powder patterns and refinement of the crystal structures were done with the GSAS and EXPGUI packages^58^.

### YH_3_ and other phases formed under hydrogen deficiency

In separate experiments, we characterised YH_3_ and YD_3_, which were the starting materials for the synthesis of higher hydrides in our study, during compression up to 180 GPa. *Hcp*-YH_3_ is the yttrium hydride with the highest hydrogen content under ambient conditions. It is a black narrow-bandgap semiconductor with a metallic lustre. At increasing pressure, *hcp*-YH_3_ undergoes a continuous phase transition at ~10–25 GPa into *fcc*-YH_3_^52,59,60^. A further pressure increase results in continuous metallisation, which is accompanied by a disappearance of the Raman spectrum at ∼80 GPa and a significant decrease in electrical resistance from ∼50 Ω at 16 GPa to ∼0.12 Ω at 81 GPa (Supplementary Fig. 8). This behaviour agrees well with previous measurements^61^. YH_3_ and YD_3_ retain the *fcc* metal sublattice upon compression up to ∼150 GPa (samples 12–13 and 26–30), in agreement with theoretical calculations^31^. However, we observed the formation of another *fcc* phase in addition to *fcc*-YH_3_(YD_3_) at pressures above ∼100 GPa during the compression of the initial YH_2.92(5)_ and YD_2.87(5)_ samples (Supplementary Fig. 6). The formation of this new phase is accompanied by the appearance of a strong Raman spectrum. Its lattice volume is smaller by ~5 Å^3^ per Y atom than that of YH_3_/YD_3_, likely indicating a reduced hydrogen content close to YH_1_/YD_1_. We did not observe this hydrogen-depleted phase in samples compressed in H_2_ or D_2_ medium (samples 25, 27–29). Thus, the formation of the *fcc*-YH_1_/YD_1_ phase is likely driven by the non-stoichiometric composition of the initial materials. A similar phenomenon was previously observed in substoichiometric LaH_2.3_ upon compression in an inert medium, and it was attributed to a disproportionation reaction into the hydrogen-enriched stoichiometric LaH_3_ and hydrogen-depleted solid solution^62^.

In addition to the *I4/mmm* YH_4_, *Im-3m* YH_6_ and *P6*_3_*/mmc* YH_9_ phases discussed in detail in the main text, we observed some unidentified impurity phases with complex X-ray powder diffraction patterns. Typical X-ray diffraction powder patterns of unidentified impurities are plotted in Supplementary Fig. 9a. Since such phases were found in samples with an evident deficiency of H_2_ (D_2_) or in the poorly heated areas of samples, their H/Y ratio is likely <9. Troyan et al.^26^ also found some new phases at pressures of 165–180 GPa and assigned them to the YH_7_ and Y_2_H_15_ hydrides. None of these phases fit the reflections from the unidentified phases observed in the present study.

In sample 28 at a lower pressure of 105 GPa, we found a new *bcc* phase with a composition close to YH_4_ (Supplementary Fig. 9b, c).

## Supplementary information

Supplementary Information

## Data Availability

The data that support the findings of this study are available from the corresponding author upon reasonable request.
