# Supplementary material for: Superconductivity up to 243 K in the yttrium-hydrogen system under high pressure
Source: Nat Commun. 2021 Aug 20;12:5075. doi: 10.1038/s41467-021-25372-2 (PMC8379216; doi:10.1038/s41467-021-25372-2)
Supplement: Supplementary file 1 — Supplementary Information [file 41467_2021_25372_MOESM1_ESM.pdf]

Superconductivity up to 243 K in the yttrium-hydrogen system under high pressure

Panpan Kong[1,7], Vasily S. Minkov[1,7], Mikhail A. Kuzovnikov[2,7], Alexander P. Drozdov[1], Stanislav P. Besedin[1], Shirin Mozaffari[3], Luis Balicas[3], Fedor Fedorovich Balakirev[4], Vitali B. Prakapenka[5], Stella Chariton[5], Dmitry A. Knyazev[6], Eran Greenberg[5] and Mikhail I. Eremets[1*]

[1]Max-Planck-Institut für Chemie, Hahn-Meitner Weg 1, Mainz 55128, Germany

[2]Institute of Solid State Physics Russian Academy of Sciences, 2 Academician Ossipyan Str., Chernogolovka, Moscow District 142432, Russia

[3]National High Magnetic Field Laboratory, Florida State University, Tallahassee, Florida 32310, USA

[4]NHMFL, Los Alamos National Laboratory, MS E536, Los Alamos, New Mexico 87545, USA

[5]Center for Advanced Radiation Sources, University of Chicago, 5640 South Ellis Avenue, Chicago, Illinois, 60637, USA

[6] Max-Planck-Institut für Mikrostrukturphysik, Weinberg 2, Halle (Saale) 06120, Germany

[7]These authors contributed equally: Panpan Kong, Vasily S. Minkov, Mikhail A. Kuzovnikov

*e-mail: m.eremets@mpic.de

Supplementary Table 1. List of samples synthesized and studied in the present work. Pressure values are estimated using the hydrogen scale[1] if the corresponding high-frequency vibron from H_2 or D_2 medium could be observed in Raman spectra (P_H) and diamond scale[2] following the shift of the stressed diamond line edge (P_D).

Sample	***Photo***	***Synthesis conditions***	***Electrical measurements***	***X-ray diffraction***
1	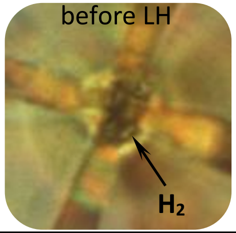	YH_3 + H_2. Pressurized to P_H=237 GPa (P_D=255 GPa) and kept for 3 weeks at room temperature. H_2 was in large excess	Two steps at T_c = 227 K (likely YH_9) T_c = 208.5 K (YH_6) Fig.4e, filled red circle and star, both outlined by the red circles	A dominant phase ***Im-3m*** YH_6: *a*=3.452(1) Å, *V*=41.1(1) Å^3; with a minor impurity of ***I4/mmm*** YH_4*:* *a*=2.616(1) Å, *c*=5.184(1) Å, *V*=35.5(1) Å^3. Supplementary Fig.2a
	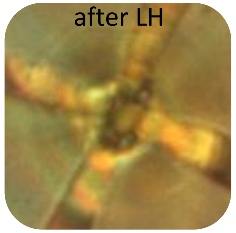	The same sample heated by a pulse laser below 700 K (no visible glowing)	T_c = 237 K Fig.4a, blue curve; Fig.4e, filled red circle	A mixture of ***P6₃/mmc*** YH_9: *a*=3.364(1) Å, *c*=5.153(1) Å, *V*=50.5(1) Å^3; and ***Im-3m*** YH_6: *a*=3.457(1) Å, *V*=41.3(1) Å^3; and traces of ***I4/mmm*** YH_4. Supplementary Fig.2b
		The same sample decompressed to P_H=215 GPa	T_c ~ 242 K Fig.4e, open red circles	n/a
	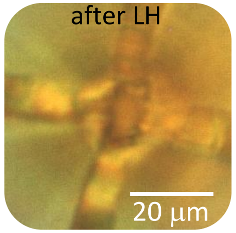	The same sample compressed to P_H=255 GPa (P_D=284 GPa) and then heated by a pulse laser at ~1000 K (weak glowing)	T_c = 235 K Fig.4e, filled red circles	A dominant phase ***P6₃/mmc*** YH_9: *a*=3.336(1) Å, *c*= 5.088(4) Å, *V*=49.1(1) Å^3; with traces of ***Im-3m*** YH_6: *a*=3.417(1) Å, *V*=39.9(1) Å^3. Fig.1c; Supplementary Fig.2c

2	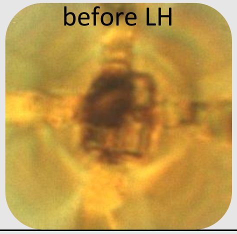	$YH_3 + H_2$. Pressurized to P_H=201 GPa (P_D=238 GPa) and kept for 1 month at room temperature. H_2 was in large excess	T_c ~ 211 K Fig.4a, black curve; Fig.4e, black star marked by red circle	A mixture of ***Im-3m*** YH_6: a=3.492(1) Å, V=42.6(1) Å^3; and ***I4/mmm*** YH_4: a=2.656(5) Å, c=5.190(10) Å, V=36.6(1) Å^3. Fig.1b; Supplementary Fig.2d
	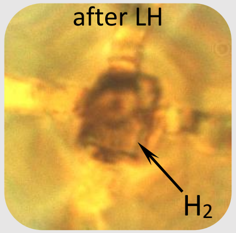	The same sample heated by a pulse laser at 2000(10) K	T_c = 243 K Fig.4a, red curve; Fig.4e, filled black circle; Fig.4d, black curve	A mixture of ***P6₃/mmc*** YH_9: a=3.406(1) Å, c=5.210(8) Å, V=52.3(1) Å^3; and ***Im-3m*** YH_6: a=3.492(1) Å, V=42.6(1) Å^3; and traces of ***I4/mmm*** YH_4. Supplementary Fig.2e
	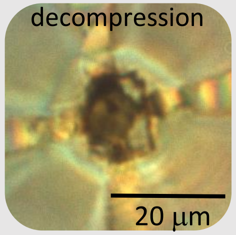	The same sample decompressed to P_H=159 GPa (P_D=189 GPa)	T_c = 220 K (three-probe measurements) Fig.4e, open black star	Almost pure ***Im-3m*** YH_6: a=3.571(1) Å, V=45.5(1) Å^3. Supplementary Fig.2f
3	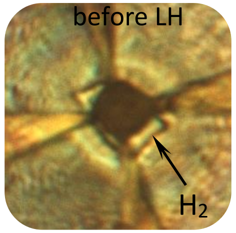 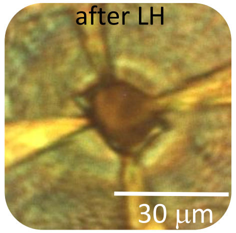	Y + H_2. Pressurized to P_H=186 GPa (P_D=228 GPa) and heated by a pulse laser at ~1500 K	T_c = 239 K Fig.4e, violet circle	A mixture of ***I4/mmm*** YH_4: a=2.666(1) Å, c=5.194(1) Å, V=36.9(1) Å^3; ***Im-3m*** YH_6: a=3.529(1) Å, V=43.9(1) Å^3; and ***P6₃/mmc*** YH_9: a=3.432(3) Å, c=5.251(3) Å, V=53.6(1) Å^3.

4	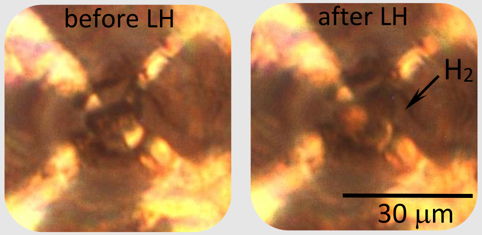	Y + H_2. Pressurized to P_H=183 GPa (P_D=189 GPa) and heated by a pulse laser at ~1500 K	T_c = 220 K<br>Fig.4c, olive curve; Fig.4e, olive star	A mixture of ***I4/mmm*** YH_4:<br>a=2.708(1) Å, c=5.195(2) Å, V=38.1(1) Å^3;<br>and ***Im-3m*** YH_6:<br>a=3.542(2) Å, V=44.4(1) Å^3;<br>and unidentified impurity(ies).<br>Fig.1a; Supplementary Fig.9a
5	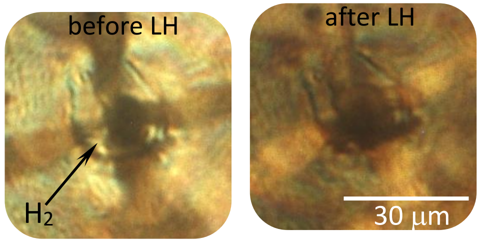	Y + H_2. Pressurized to P_H=160 GPa (P_D=185 GPa) and heated by a pulse laser at ~1500 K	T_c = 214 K<br>(three-probe measurement)<br>Fig.4e, cerulean star; Fig.5a	A dominant phase ***Im-3m*** YH_6:<br>a=3.570(5) Å, V=45.5(1) Å^3;<br>with impurities of ***I4/mmm*** YH_4 and unidentified phase(s).<br>Supplementary Fig.9a
6	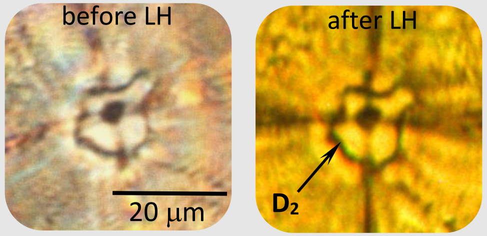	YD_3 + D_2. Pressurized to P_H=202 GPa (P_D=209 GPa) and kept for 16 days at room temperature. D_2 was in large excess	T_c = 165 K<br>Fig.4b, black curve; Fig.4f, blue stars marked by red circles	A mixture of ***I4/mmm*** YD_4:<br>a=2.685(10) Å, c=5.195(10) Å, V=37.4(1) Å^3;<br>and ***Im-3m*** YD_6:<br>a=3.499(5) Å, V=42.8(1) Å^3.<br>Supplementary Fig.3a
		The same sample kept for 140 days and heated by a pulse laser at 1800(50) K. P_H=205 GPa (P_D=212 GPa)	T_c = 172 K<br>Fig.4b, red curve; Fig.4f, blue circle; Fig.5b	A dominant phase ***P6₃/mmc*** YD_9:<br>a=3.404(5) Å, c=5.244(5) Å, V=52.6(1) Å^3<br>and ***Im-3m*** YD_6:<br>a=3.507(5) Å, V=43.1(1) Å^3.<br>Supplementary Fig.3b
7	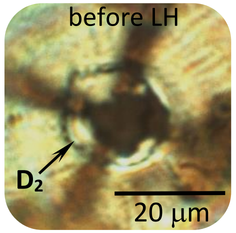	YD_3 + D_2. Pressurized to P_H=173 GPa (P_D=194 GPa) and heated by a pulse laser at ~1500 K	T_c = 168 K<br>Fig.4c, magenta curve; Fig.4f, magenta stars	***Im-3m*** YD_6:<br>a=3.552(1) Å, V=44.8(1) Å^3;<br>***I4/mmm*** YD_4:<br>a=2.712(1) Å, c=5.190(1) Å, V=38.2(1) Å^3.
		The same sample pressurized to P_D=212 GPa and repeatedly heated by a pulse laser at ~1500 K	T_c = 172 K (likely YD_9)<br>Fig.4f, magenta circle	n/a

8	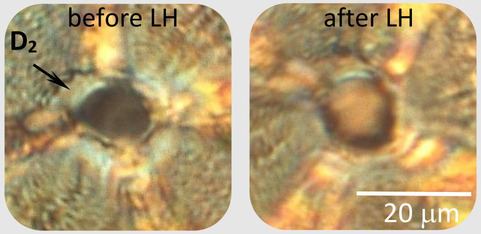	Y + D_2. Pressurized to P_D=195 GPa and heated by a pulse laser at ~1500 K. D_2 was in deficiency	T_c = 166 K (likely YD_6) Fig.4f, orange stars	n/a
		Compressed to P_D=330 GPa	T_c = 156 K (likely YD_6) Fig.4f, orange stars	n/a
9	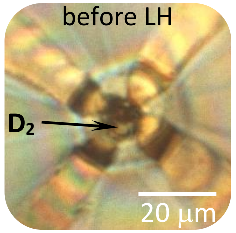	YD_3 + D_2. Pressurized to P_H=227 GPa (P_D=242 GPa) and kept for 2 weeks at room temperature. D_2 was in large excess	T_c = 166 K (likely YD_6) Fig.4f, red star marked by red circle	n/a
10	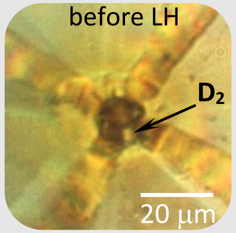	YD_3 + D_2. Pressurized to P_H=244 GPa (P_D=262 GPa) and kept for 3 weeks at room temperature. D_2 was in large excess	T_c = 160 K Fig.4f, green star marked by red circle	A dominant phase ***I4/mmm*** YD_4: a=2.645(5) Å, c=5.135(5) Å, V=35.9(1) Å^3; and ***Im-3m*** YD_6: a=3.455(5) Å, V=41.3(1) Å^3; and a minor unidentified impurity(ies).
		The same sample heated by a pulse laser at 1700(50) K	n/a	A mixture of ***P6₃/mmc*** YD_9: a=3.392(5) Å, c=5.180(5) Å, V=51.6(1) Å^3 and ***Im-3m*** YD_6: a=3.478(5) Å, V=42.1(1) Å^3.
11	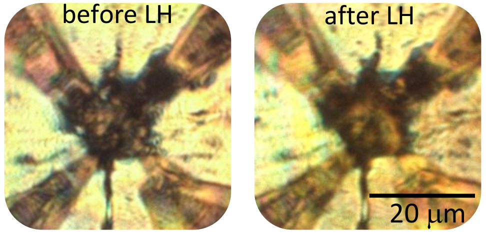	YH_3 + NH_3BH_3. Pressurized to P_D=250 GPa and heated by a pulse laser at 1850(50) K	Metallic behavior on cooling with R of ~0.07 Ω at 78 K Supplementary Fig.4a and 8a	A mixture of ***I4/mmm*** YH_4*:* a=2.620(1) Å, c=5.161(2) Å, V=35.4(1) Å^3; and distorted ***Fm-3m*** YH_3: a=4.17(1) Å, V=72.8(3) Å^3. Supplementary Fig.4b

12	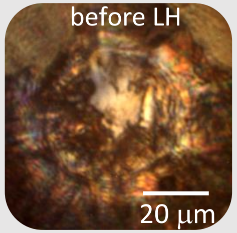	YH_3. Pressurized to P_D=130 GPa and heated by a pulse laser at ~1000 K	Metallic behavior on cooling with R of ~0.04 Ω at 5 K Supplementary Fig.8a	A mixture of ***Fm-3m*** YH_3: a=4.373(6) Å, V=83.6(4) $Å^3$; and ***Fm-3m*** YH_1: a=3.986(2) Å, V=63.4(1) $Å^3$.
13	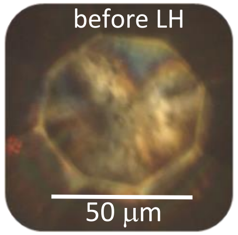	YD_3. Pressurized to P_D=135 GPa and heated by a pulse laser at ~1000 K	Metallic behavior on cooling with R of ~0.03 Ω at 5 K Supplementary Fig.4a and 8	A mixture of ***Fm-3m*** YD_3: a=4.332(1) Å, V=81.3(1) $Å^3$; and ***Fm-3m*** YD_1: a=3.975(1) Å, V=62.8(1) $Å^3$. Supplementary Fig.6b
14	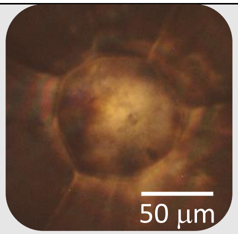	YD_3. Pressurized to P_D=180 GPa	Metallic behavior on cooling with R of ~0.5 Ω at 5 K	A mixture of distorted ***Fm-3m*** YD_3: a=4.279(2) Å, V=78.3(2) $Å^3$; and ***Fm-3m*** YD_1: a=3.876(1) Å, V=58.2(1) $Å^3$.
15	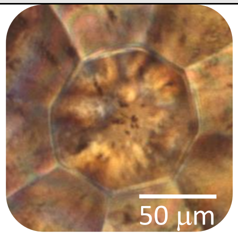	YH_3. Pressurized to P_D=170 GPa	Metallic behavior on cooling with R of ~0.05 Ω at 5 K Supplementary Fig.4a and 8a	A mixture of distorted ***Fm-3m*** YH_3: a=4.286(2) Å, V=78.8(1) $Å^3$; and ***Fm-3m*** YH_1: a=3.901(1) Å, V=59.4(1) $Å^3$. Supplementary Fig.6a

16	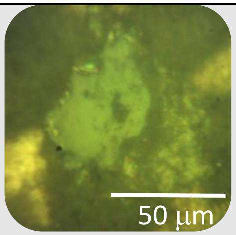	YD_3. Pressurized to P_D=61 GPa	Metallic behavior on cooling with R of ~0.17 Ω at 5 K Supplementary Fig.8a	n/a
17	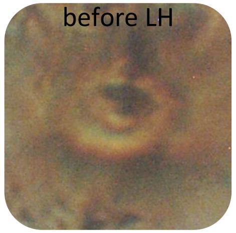 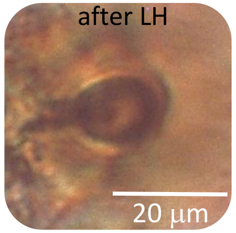	YH_3 + NH_3BH_3. Pressurized to P_D=410 GPa and heated by a pulse laser at 2250(10) K	n/a	Single phase of ***P6₃/mmc*** YH_9: *a*=3.234(1) Å, *c*=4.915(1) Å, *V*=44.5(1) Å^3. Fig.3b
18	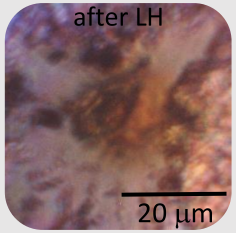	YH_3 + NH_3BH_3. Pressurized to P_D=395 GPa and heated by a pulse laser at 1815(5) K	n/a	A mixture of ***Im-3m*** YH_6: *a*=3.316(5) Å, *V*=36.5(1) Å^3; and ***I4/mmm*** YH_4: *a*=2.458(5) Å, *c*=5.140(5) Å, *V*=31.1(1) Å^3.
		The same sample heated several times by a pulse laser at 1830(5) K	n/a	A mixture of ***P6₃/mmc*** YH_9: *a*=3.254(5) Å, *c*=4.914(5) Å, *V*=45.1(1) Å^3; and ***Im-3m*** YH_6: *a*=3.316(5) Å, *V*=36.5(1) Å^3.
19	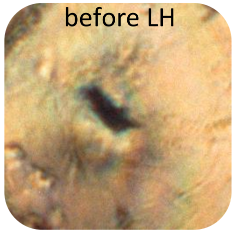 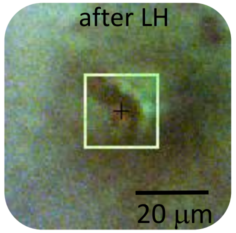	YH_3 + NH_3BH_3. Pressurized to P_D=325 GPa	n/a	Distorted ***Fm-3m*** YH_3: *a*=4.05(5) Å, *V*=66(2) Å^3.
		The same sample compressed to P_D=341 GPa and heated by a pulse laser several times at 1725(5) K	n/a	A mixture of ***P6₃/mmc*** YH_9: *a*=3.292(5) Å, *c*=5.022(5) Å, *V*=47.1(1) Å^3; ***Im-3m*** YH_6: *a*=3.370(5) Å, *V*=38.3(1) Å^3; and ***Fm-3m*** YH_3: a=3.914(5) Å, V=60.0(1) Å^3; and a minor unidentified impurity(ies).

20	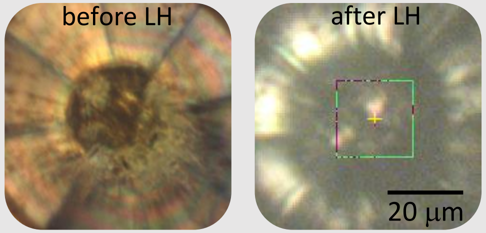	$YH_3 + NH_3BH_3$. Pressurized to P_D=335 GPa and heated by a pulse laser at 2060(10) K	n/a	A mixture of ***Im-3m*** YH_6:<br>*a*=3.370(5) Å, *V*=38.3(1) Å^3;<br>and ***I4/mmm*** YH_4*:*<br>*a*=2.536(5) Å, *c*=5.126(5) Å, *V*=33.0(1) Å^3.
21	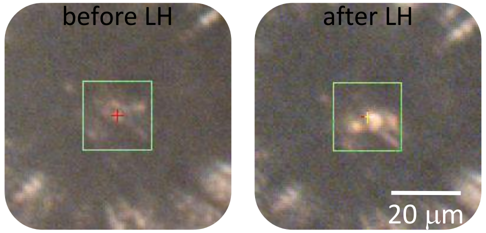	$YH_3 + NH_3BH_3$. Pressurized to P_D=332 GPa and heated by a pulse laser at 1890(5) K	n/a	A mixture of ***Im-3m*** YH_6:<br>*a*=3.377(5) Å, *V*=38.5(1) Å^3;<br>and ***I4/mmm*** YH_4*:*<br>*a*=2.540(5) Å, *c*=5.120(5) Å, *V*=32.9(1) Å^3;<br>with minor amounts of ***$P6_3/mmc$*** YH_9:<br>*a*=3.300(5) Å, *c*=5.015(5) Å, *V*=47.3(1) Å^3;<br>and ***Fm-3m*** YH_3:<br>*a*=4.008(5) Å, *V*=64.4(1) Å^3;<br>and unidentified phase(s).
		Decompressed to P_D=223 GPa	n/a	***I4/mmm*** YH_4*:*<br>*a*=2.645(5) Å, *c*=5.100(5) Å, *V*=35.7(1) Å^3.<br>Supplementary Table 2
22	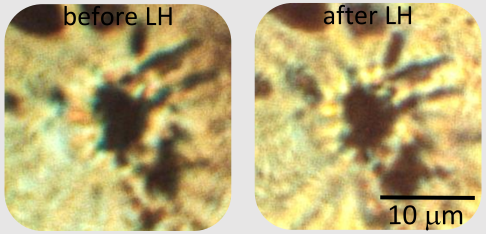	$YH_3 + NH_3BH_3$. Pressurized to P_D=235 GPa (*P*=231 GPa according to MgO gasket)	n/a	Distorted ***Fm-3m*** YH_3:<br>*a*=4.17(1) Å, *V*=72.5(10) Å^3.
		The same sample heated by a pulse laser at 2275(5) K	n/a	A mixture of ***Im-3m*** YH_6:<br>*a*=3.458(5) Å, *V*=41.3(1) Å^3;<br>and ***I4/mmm*** YH_4*:*<br>*a*=2.623(5) Å, *c*=5.168(5) Å, *V*=35.6(1) Å^3<br>and ***$P6_3/mmc$*** YH_9:<br>*a*=3.379(5) Å, *c*=5.135(5) Å, *V*=50.8(1) Å^3;<br>and minor unidentified impurity(ies).
23	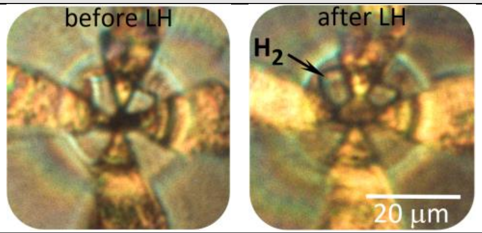	$Y + H_2$. Pressurized to P_H=187 GPa (P_D=214 GPa) and heated by a pulse laser at 2150(50) K	n/a	***$P6_3/mmc$*** YH_9:<br>*a*=3.428(5) Å, *c*=5.262(5) Å, *V*=53.6(1) Å^3;<br>and unidentified phase(s).

24	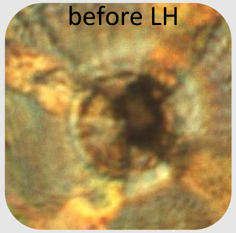 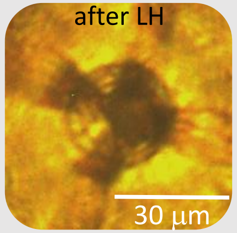	$YD_3 + D_2$. Pressurized to P_H=175 GPa and heated by a pulsed laser at ~1500 K	n/a	A dominant phase ***Im-3m*** YD_6:<br>a=3.536(1) Å, V=44.2(1) Å^3;<br>***I4/mmm*** YD_4:<br>a=2.722(5) Å, c=5.192(5) Å, V=38.5(2) Å^3<br>and unidentified phase(s).
		Decompressed to P_D=135 GPa	n/a	***I4/mmm*** YD_4:<br>a=2.773(5) Å, c=5.212(5) Å, V=40.1(2) Å^3.<br>Supplementary Table 3
25	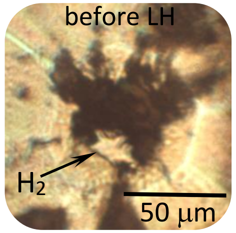 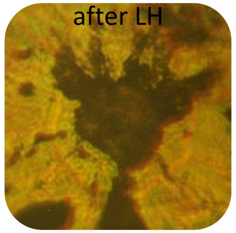	$YH_3 + H_2$. Pressurized to P_H=150 GPa (P_D=155 GPa) and heated by a pulse laser at 1800(50) K. H_2 was in deficiency	n/a	Unidentified phase(s);<br>and remains of ***Fm-3m*** YH_3:<br>a=4.284(3) Å, V=78.6(2) Å^3.<br>Supplementary Fig.9a
26	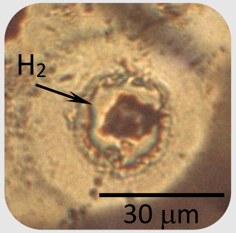	Y + H_2. Pressurized to P_D=17 GPa	n/a	***Fm-3m*** YH_3:<br>a=4.971(10) Å, V=122.8(3) Å^3.<br>Supplementary Fig.1a
		The same sample pressurized to P_D=143 GPa (according to MgO gasket)	n/a	***Fm-3m*** YH_3:<br>a=4.367(10) Å, V=83.3(3) Å^3.<br>Supplementary Table 4
27	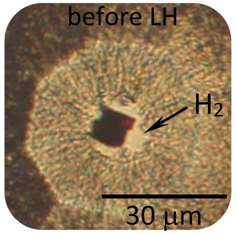	Y + H_2. Compressed to P_D=23 GPa	n/a	***Fm-3m*** YH_3:<br>a=4.930(10) Å, V=119.8(3) Å^3.<br>Supplementary Fig.1b
		The same sample compressed to P=140 GPa (according to MgO gasket) and heated by a pulse laser at ~2600 K	n/a	Unidentified phase(s);<br>and remains of ***Fm-3m*** YH_3:<br>a=4.397(10) Å, V=85.0(3) Å^3.<br>Supplementary Table 5

28	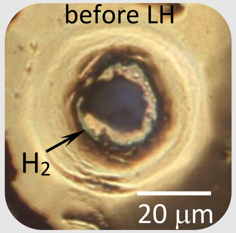	Y + H_2. Pressurized to P_H=105 GPa (P_D=110 GPa)	n/a	***Fm-3m*** YH_3:<br>a=4.452(1) Å, V=88.2(1) Å^3.<br>Supplementary Fig.9b
		The same sample heated by a pulse laser several times at 1500(50) K	n/a	A mixture of ***Fm-3m*** YH_3:<br>a=4.459(1) Å, V=88.7(1) Å^3;<br>and ***Im-3m*** YH_4:<br>a=3.564(1) Å, V=45.2(1) Å^3.<br>Supplementary Fig.9c
		The same sample pressurized to P_D=130 GPa and heated by a pulse laser several times at 2600(50) K	n/a	A mixture of ***Fm-3m*** YH_3:<br>a=4.388(1) Å, V=84.5(1) Å^3;<br>and unidentified phase(s).<br>Supplementary Fig.9a
29	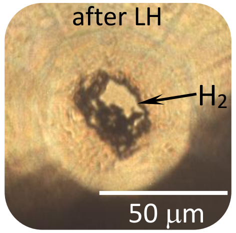	YH_3 + H_2. Pressurized to P_H=120 GPa (P_D=120 GPa) and slightly heated by a pulse laser at ~700 K. H_2 was in large excess	n/a	Mainly ***Fm-3m*** YH_3:<br>a=4.421(5) Å, V=86.4(1) Å^3;<br>and traces of unidentified impurity(ies).
30	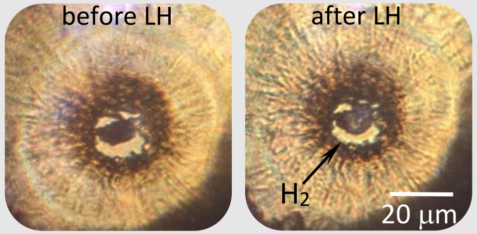	Y + H_2. Pressurized to P_H=85 GPa (P_D=90 GPa) and heated by a pulsed laser at ~1000 K	n/a	***Fm-3m*** YH_3:<br>a=4.519(5) Å, V=92.3(1) Å^3.
31	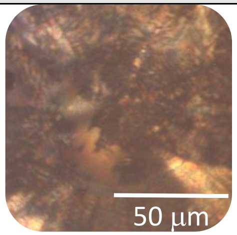	YD_3. Pressurized to P_D=168 GPa at ~100 K and then warmed	n/a	Distorted ***Fm-3m*** YD_3 phase:<br>a=4.28(1) Å, V=78.4(10) Å^3;<br>and traces of ***Fm-3m*** YD_1.

Supplementary Table 2. Changes in lattice parameters of *Fm-3m* YH_3, *I4/mmm* YH_4, and *Im-3m* YH_6 in sample 21 upon decompression. Pressure values are estimated using the diamond scale,[2] P_D.

Phase / P_D (GPa)	***Im-3m*** YH_6		***I4/mmm*** YH_4			***Fm-3m*** YH_3	
	a (Å)	*V* (Å^3)	*a* (Å)	*c* (Å)	*V* (Å^3)	*a* (Å)	*V* (Å^3)
332	3.377(5)	38.5(1)	2.540(5)	5.120(5)	32.9(1)	4.008(5)	64.4(1)
323	3.370(1)	38.3(1)	2.547(5)	5.137(5)	33.3(1)	4.023(5)	65.1(1)
315	3.400(1)	39.3(1)	2.564(5)	5.137(5)	33.8(1)	4.029(5)	65.4(1)
305	3.380(1)	38.6(1)	2.564(5)	5.143(5)	33.8(1)	4.038(5)	65.8(1)
282	*	*	2.588(5)	5.161(5)	34.6(1)	*	*
251	*	*	2.609(5)	5.184(5)	35.3(1)	*	*
223	*	*	2.645(5)	5.100(5)	35.7(1)	*	*

* cannot be refined because reflections are strongly overlapped with those from other phases.

Supplementary Table 3. Changes in lattice parameters of *I4/mmm* YD_4 and *Im-3m* YD_6 in sample 24 with a variation in the pressure. Pressure values are estimated using the diamond scale,[2] P_D.

Phase / P_D (GPa)	***Im-3m*** YD_6		***I4/mmm*** YD_4		
	a (Å)	*V* (Å^3)	*a* (Å)	*c*(Å)	*V* (Å^3)
175	3.536(1)	44.2(1)	2.722(5)	5.192(5)	38.5(2)
186	3.519(1)	43.6(1)	2.707(5)	5.185(5)	38.0(2)
179	3.529(1)	43.9(1)	2.716(5)	5.196(5)	38.3(2)
173	3.537(1)	44.3(1)	2.722(5)	5.195(5)	38.5(2)
165	3.542(1)	44.4(1)	2.731(5)	5.205(5)	38.8(2)
161	3.552(1)	44.8(1)	2.739(5)	5.200(5)	39.0(2)
155	3.556(1)	45.0(1)	2.747(5)	5.214(5)	39.3(2)
152	3.564(1)	45.3(1)	2.755(5)	5.203(5)	39.5(2)
147	3.575(1)*	45.7(1)*	2.743(5)	5.228(5)	39.3(2)
135	disappeared		2.773(5)	5.212(5)	40.1(2)

* the phase becomes distorted

Supplementary Table 4. Changes in lattice parameters of *Fm-3m* YH_3 in sample 26 upon pressurizing. Pressure values are estimated using the diamond scale[2] at 17 GPa and the equation of state of MgO[3] (gasket) during pressurizing.

Phase / P (GPa)	***Fm-3m*** YH_3	
	a (Å)	*V* (Å^3)
17	4.971(10)	122.8(3)
75	4.600(10)	97.3(3)
90	4.517(10)	92.2(3)
112	4.442(10)	87.6(3)
125	4.398(10)	85.1(3)
143	4.367(10)	83.3(3)

Supplementary Table 5. Changes in lattice parameters of *Fm-3m* YH_3 in sample 27 upon pressurizing. Pressure values are estimated using the diamond scale[2] at 23 GPa and the equation of state of MgO[3] (gasket) upon further pressurizing.

Phase / P (GPa)	***Fm-3m*** YH_3	
	a (Å)	*V* (Å^3)
23	4.930(10)	119.8(3)
51	4.779(10)	109.1(3)
64	4.689(10)	103.1(3)
70	4.653(10)	100.8(3)
93	4.545(10)	93.9(3)
107	4.485(10)	90.2(3)
119	4.448(10)	88.0(3)
125	4.424(10)	86.6(3)
133	4.413(10)	85.9(3)
140	4.397(10)	85.0(3)

Supplementary Table 6. Fitting parameters of the Vinet[4] equation of state for *Fm-3m* YH_1/YD_1, *Fm-3m* YH_3/YD_3, *I4/mmm* YH_4/YD_4, *Im-3m* YH_6/YD_6 and *P6₃/mmc* YH_9/YD_9 yttrium hydrides studied at present. We assumed the pressure *P* as an independent variable and numerically solved the Vinet equation with respect to *V* for all values of the fitting parameters V_0 and B_0 and experimental *P* values using a value of $B'_0 = 4$ for the bulk modulus pressure derivative typical of many metals and hydrides. The resulting fits are shown by the solid curves in Figure 2a.

Compound	Pressure range, GPa	V_0 (Å^3/M atom)	B_0 (GPa)	B'_0
Fm-3m YH_1/YD_1	130-180	23.2(15)	163(50)	4(fixed)
Fm-3m YH_3/YD_3	17-341	36.0(10)	91(15)	4(fixed)
I4/mmm YH_4/YD_4	135-395	26.7(7)	274(40)	4(fixed)
Im-3m YH_6/YD_6	147-395	31.3(10)	264(50)	4(fixed)
P6₃/mmc YH_9/YD_9	186-410	36.6(10)	326(40)	4(fixed)

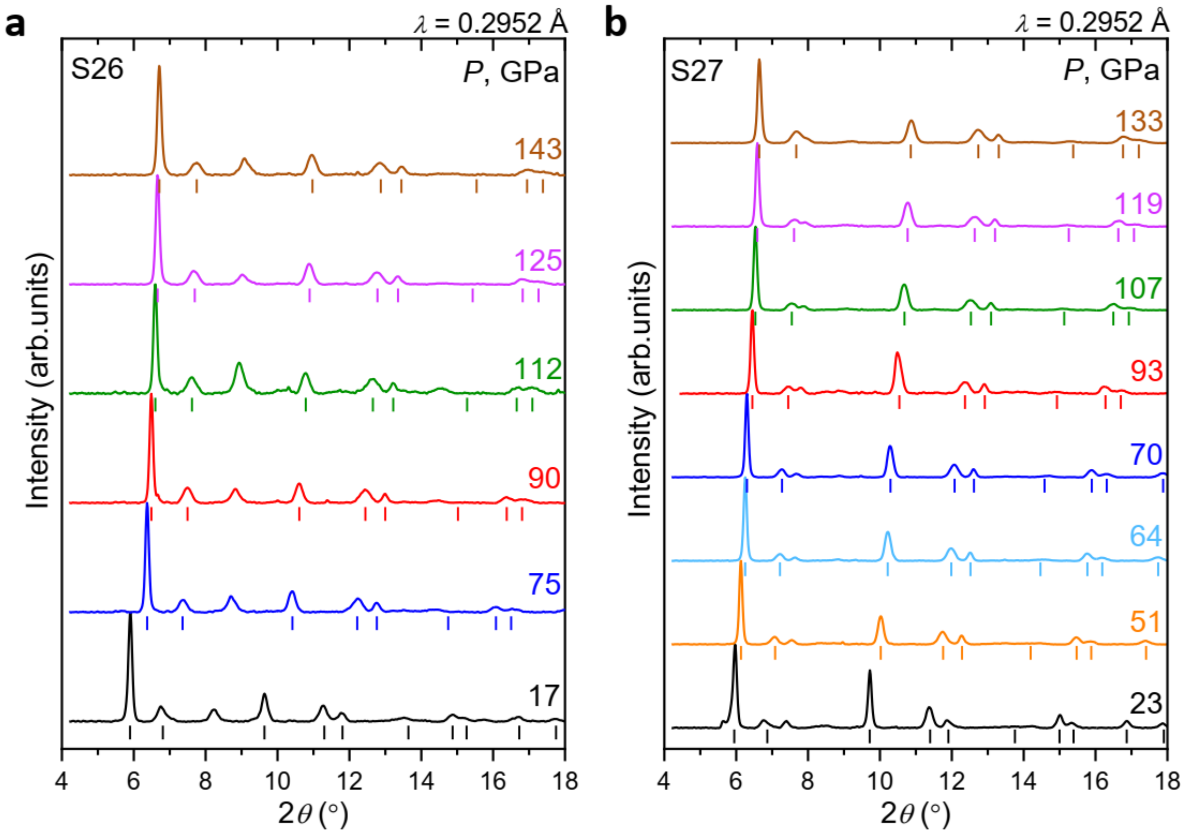

Supplementary Figure 1 X-ray powder diffraction patterns for the *Fm-3m* YH_3 phase in samples 26 and 27 synthesized by exposing Y to H_2 at room temperature.

a, **b** X-ray powder diffraction patterns show the *Fm-3m* phase of YH_3 in samples 26 and 27, respectively, at several pressures. Pressure values were estimated from the MgO gasket using the equation of state of MgO.[3] The ticks below each X-ray diffraction pattern correspond to the calculated peak positions for the *Fm-3m* phase. The refined lattice parameters are summarized in Supplementary Tables 1, 4 and 5.

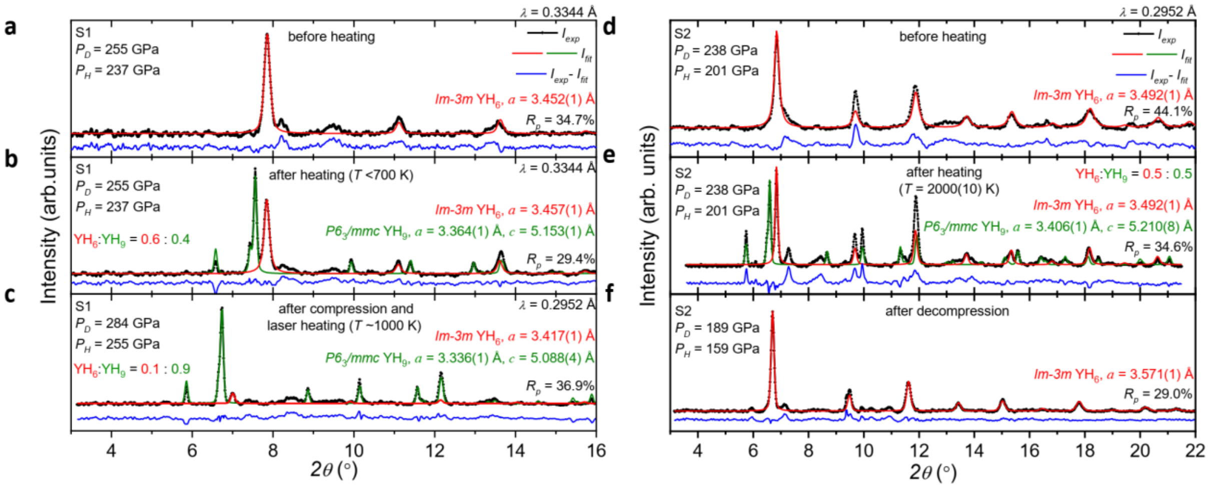

Supplementary Figure 2 X-ray powder diffraction patterns for samples 1 and 2.
a *Im-3m* YH_6 phase in sample 1 synthesized by exposing YH_3 to H_2 at P_H=237 GPa (P_D=255 GPa) for 3 weeks at room temperature. **b** The *P6$_3$/mmc* YH_9 phase formed after subtle laser heating (T<700 K) in sample 1. **c** Further conversion of YH_6 into YH_9 resulted from the subsequent pressure increase to P_H=255 GPa (P_D=284 GPa) and laser heating at T ~1000 K. **d** The *Im-3m* YH_6 phase in sample 2 formed after exposing YH_3 to H_2 at P_H=201 GPa (P_D=238 GPa) for 4 weeks at room temperature. **e** The formation of the *P6$_3$/mmc* YH_9 phase after laser heating (T=2000(10) K) in sample 2. **f** The *Im-3m* YH_6 phase in sample 2 at P_H=159 GPa (P_D=189 GPa) resulted from the decomposition of YH_9 on decompression. Red and green curves indicate the contributions of the *Im-3m* YH_6 and *P6$_3$/mmc* YH_9 phases, respectively. Black circles and blue curves correspond to the experimental data and residues, respectively.

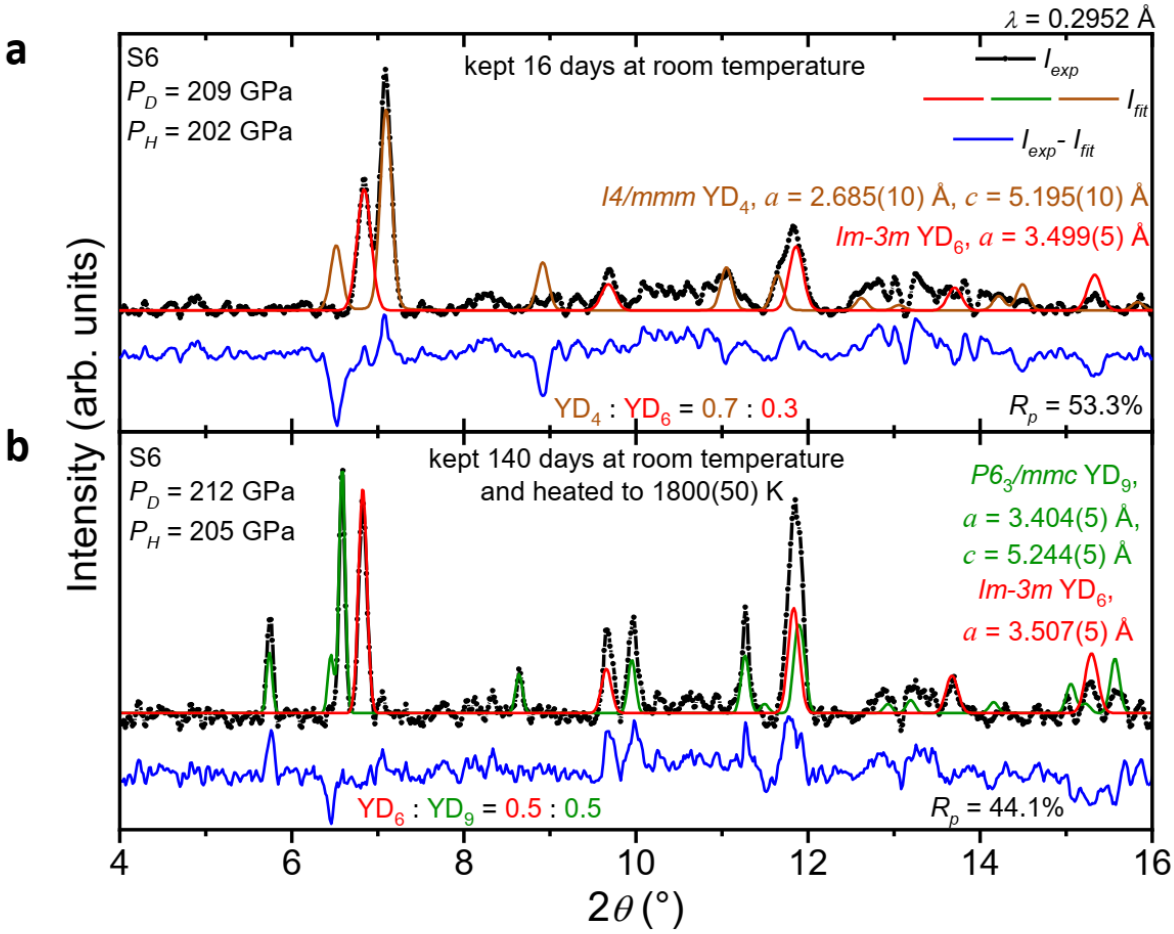

Supplementary Figure 3 X-ray powder diffraction patterns for sample 6 before and after laser heating.

a X-ray powder diffraction pattern shows the *Im-3m* YD_6 and *I4/mmm* YD_4 phases in sample 6 after exposing YD_3 to D_2 at P_H=202 GPa (P_D=209 GPa) for 16 days at room temperature. **b** X-ray powder diffraction pattern illustrating the formation of the $P6_3/mmc$ YD_9 phase after storing sample 6 for 140 days at room temperature and subsequent laser heating at 1800(50) K. Red, brown and green curves indicate the contributions of the *Im-3m* YD_6, *I4/mmm* YD_4 and $P6_3/mmc$ YH_9 phases, respectively. Black circles and blue curves correspond to the experimental data and residues, respectively.

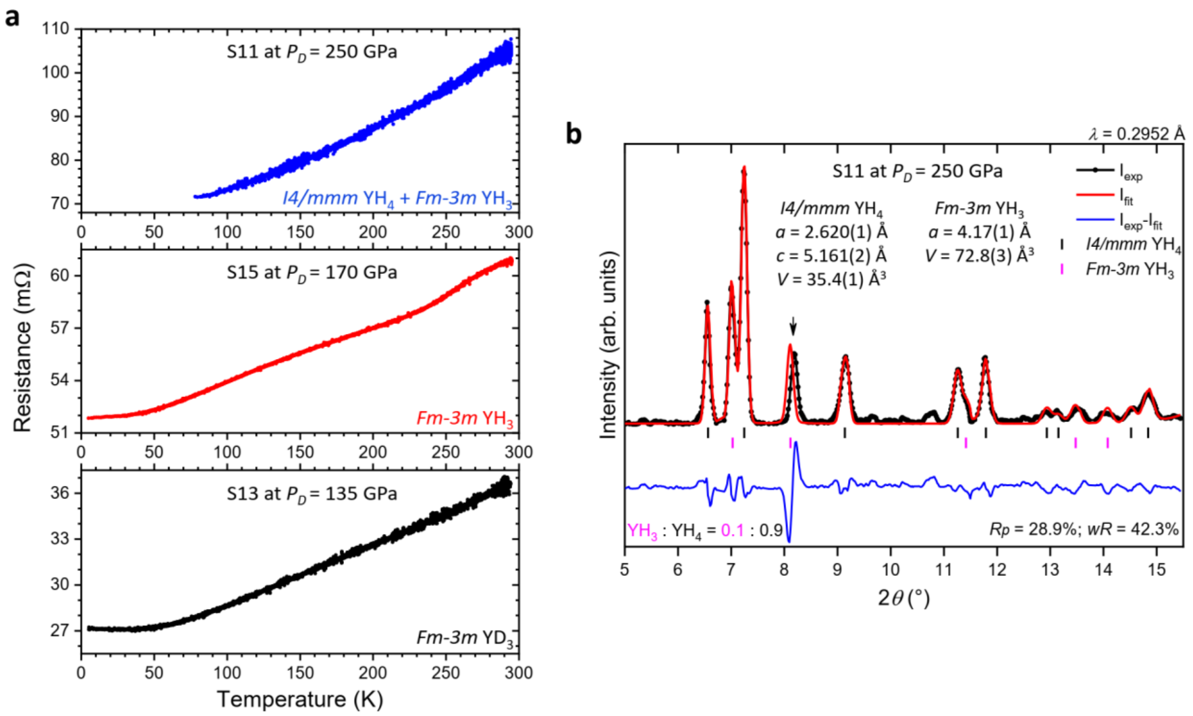

Supplementary Figure 4 Electrical resistance measurements for *Fm-3m* YH_3 and *I4/mmm* YH_4 at high pressures.

a Temperature dependence of the resistance for the mixture of *I4/mmm* YH_4 and *Fm-3m* YH_3 at P_D=250 GPa (sample 11, blue curve), *Fm-3m* YH_3 at P_D=170 GPa (sample 15, red curve), and *Fm-3m* YD_3 at P_D=135 GPa (sample 13, black curve). All three curves demonstrate the typical behaviour of a metal. **b** The X-ray powder diffraction pattern of sample 11 at P_D=250 GPa and the Rietveld refinement of the mixture of *I4/mmm* YH_4 and *Fm-3m* YH_3 phases with a refined weight ratio of 0.9:0.1. The structural distortions in the *Fm-3m* lattice of YH_3 affect the (200) reflection, marked by black arrow. Black circles, red and blue curves correspond to the experimental data, Rietveld fit and residues, respectively. Black and magenta ticks indicate the calculated peak positions for the *I4/mmm* YH_4 and *Fm-3m* YH_3 phases, respectively.

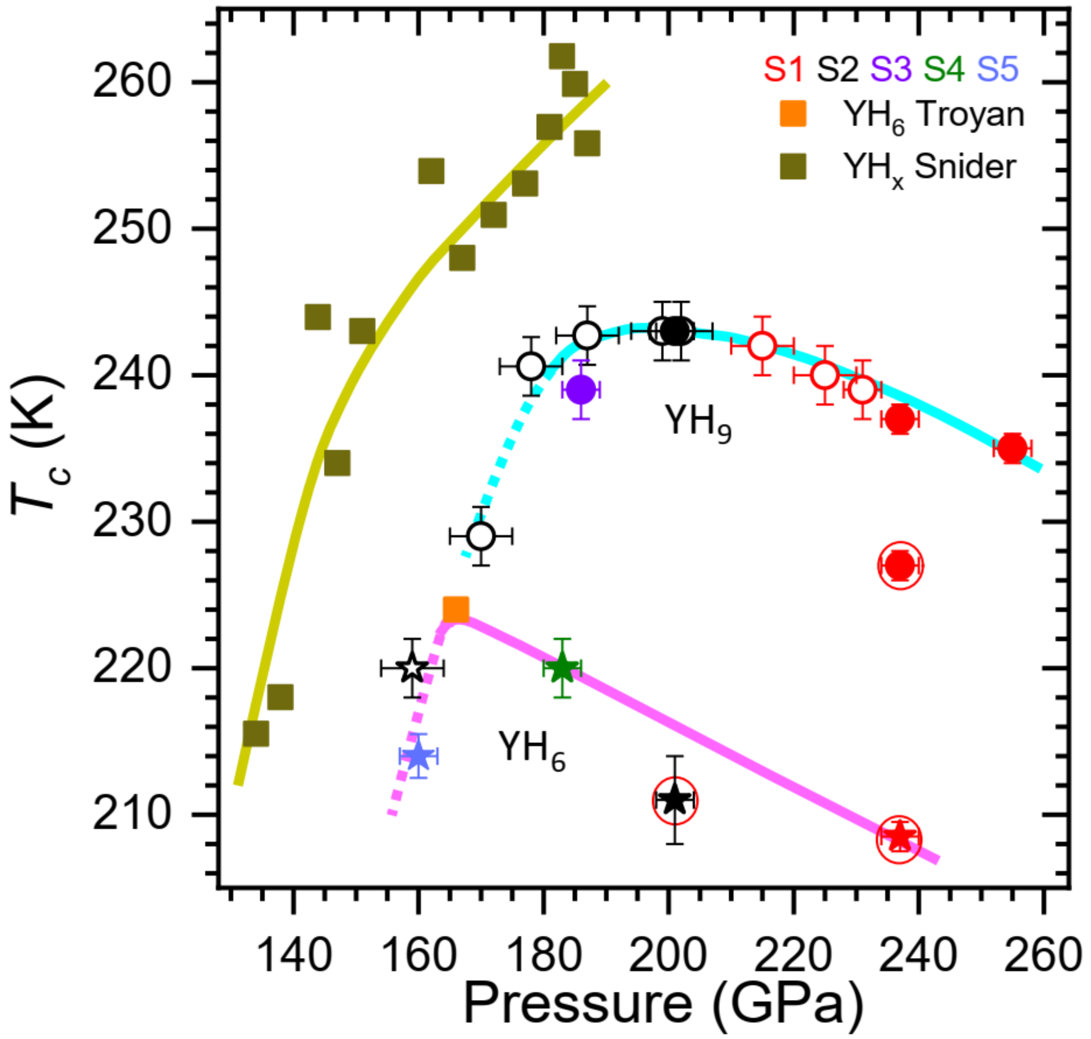

Supplementary Figure 5 Summarized $T_c(P)$ dependencies measured in the yttrium-hydrogen system at high pressures. The pressure dependence of T_c for superconducting transitions in the *Im-3m* YH_6 (stars) and *$P6_3/mmc$* YH_9 (circles) phases measured in the present study in comparison with the data published by Troyan et al.[5] (orange square) and Snider et al.[6] (dark yellow squares). Different colours represent different samples. Open symbols are the data obtained on decompression. Symbols marked by red circles are the data for the unheated samples. Curves are the guides for the eye. The data reported in Ref.[6] have systematically higher T_cs than the data collected in the present study and Ref.[5]

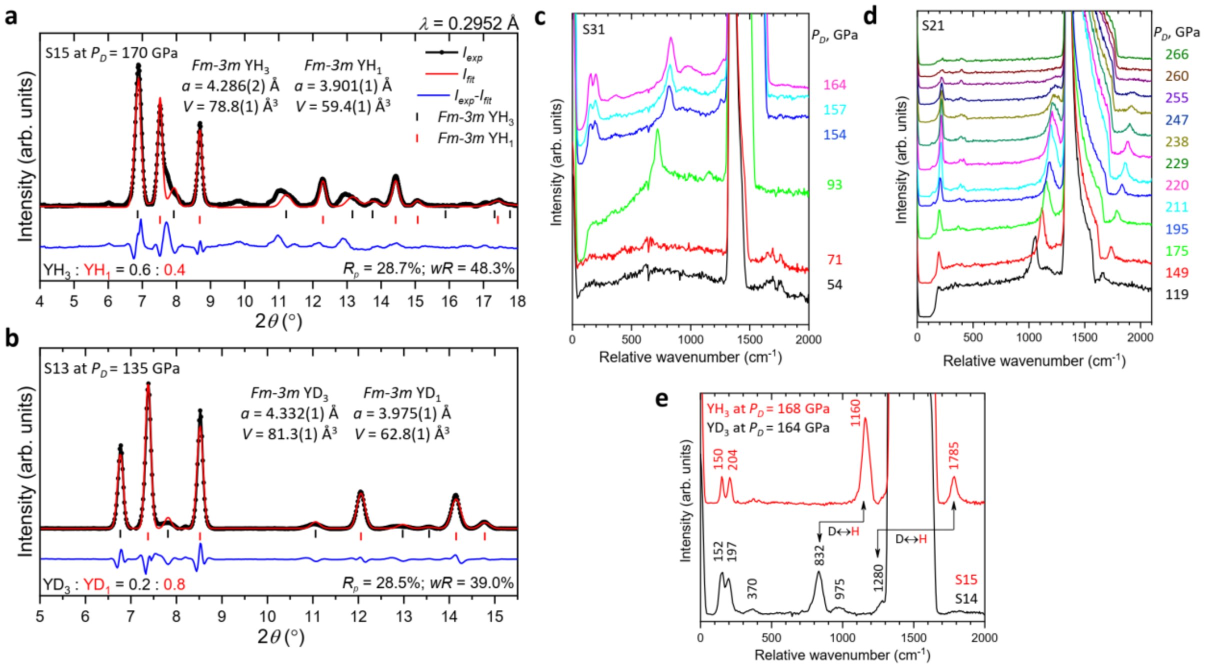

Supplementary Figure 6 Formation of the new *Fm-3m* YH_1 and YD_1 phases upon compression of $YH_{2.92(5)}$ and $YD_{2.87(5)}$ above 90 GPa.

a, b X-ray powder diffraction patterns with Rietveld refinement showing the appearance of new *Fm-3m* YH_1/YD_1 phases in sample 15 at P_D=170 GPa and sample 13 at P_D=135 GPa, respectively. Black circles, red and blue curves correspond to the experimental data, Rietveld fits and residues, respectively. Black and red ticks indicate the calculated peak positions for the *Fm-3m* YH_3 and *Fm-3m* YH_1 phases, respectively. The formation of the *Fm-3m* YH_1/ YD_1 phases after metallization of YH_3/YD_3 near 80 GPa is accompanied by the appearance of new intensive modes in the Raman spectra for samples 31, (**c**) and 21, (**d**). The Raman spectrum from the mixture of distorted *Fm-3m* YH_3 and *Fm-3m* YH_1 in sample 21 disappears at approximately 260 GPa upon further compression (**d**). Some Raman modes are associated with the Y-H stretching vibrations and shift to lower wavenumbers at approximately $\sqrt{2}$ for deuteride (**e**).

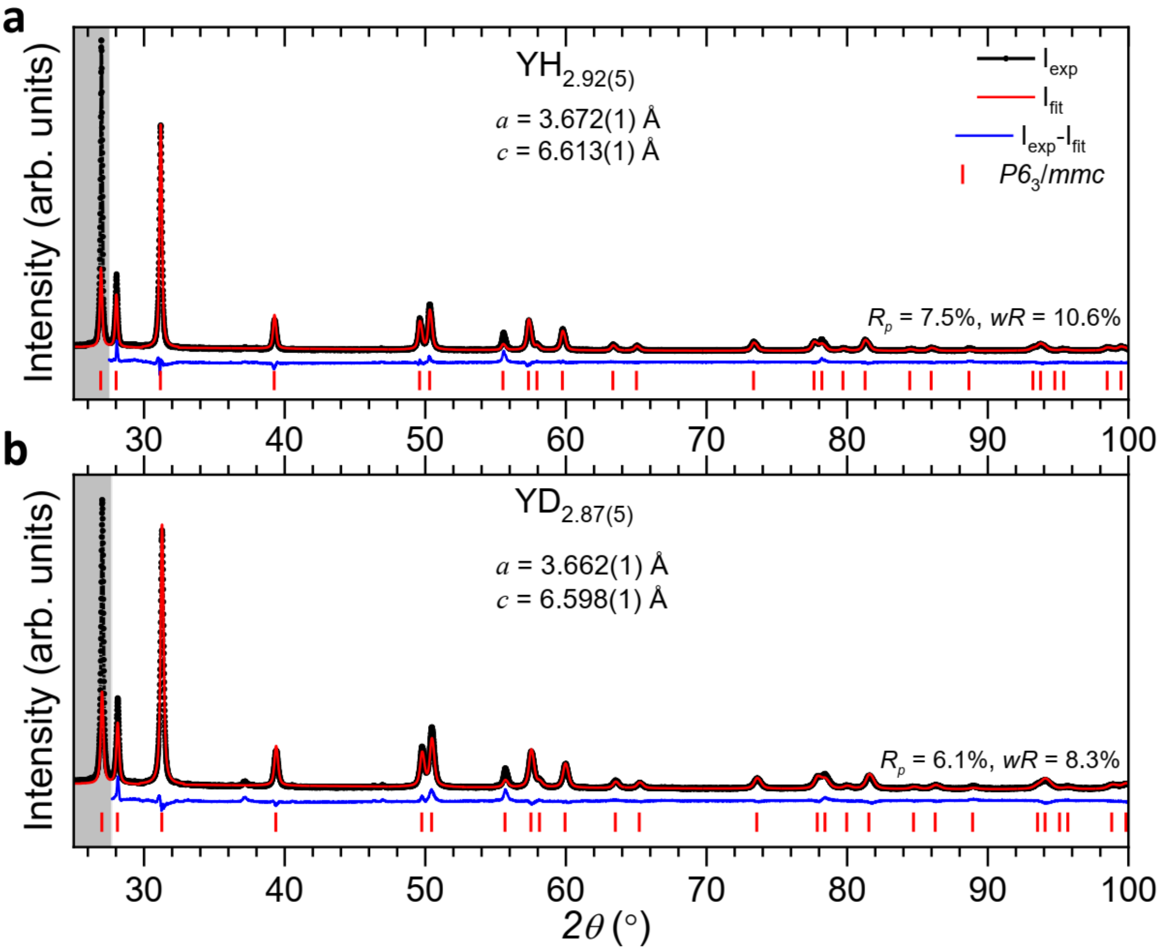

Supplementary Figure 7 X-ray powder diffraction patterns of specially synthesized *hcp*-$YH_{2.92(5)}$ (**a**) and *hcp*-$YD_{2.87(5)}$ (**b**) samples, which were used as the initial reactants for the high-pressure synthesis of the higher hydrides (deuterides) in the DACs. The X-ray powder diffraction patterns were measured at ambient pressure and temperature in an inert atmosphere with Cu K_α radiation. The first (002) reflection marked by the grey-shaded region was excluded from the Rietveld refinement because of strong texture effects in the samples. Black circles, red and blue curves correspond to the experimental data, Rietveld fits and residues, respectively. Red ticks indicate the calculated peak positions for $P6_3/mmc$ YH_3/YD_3.

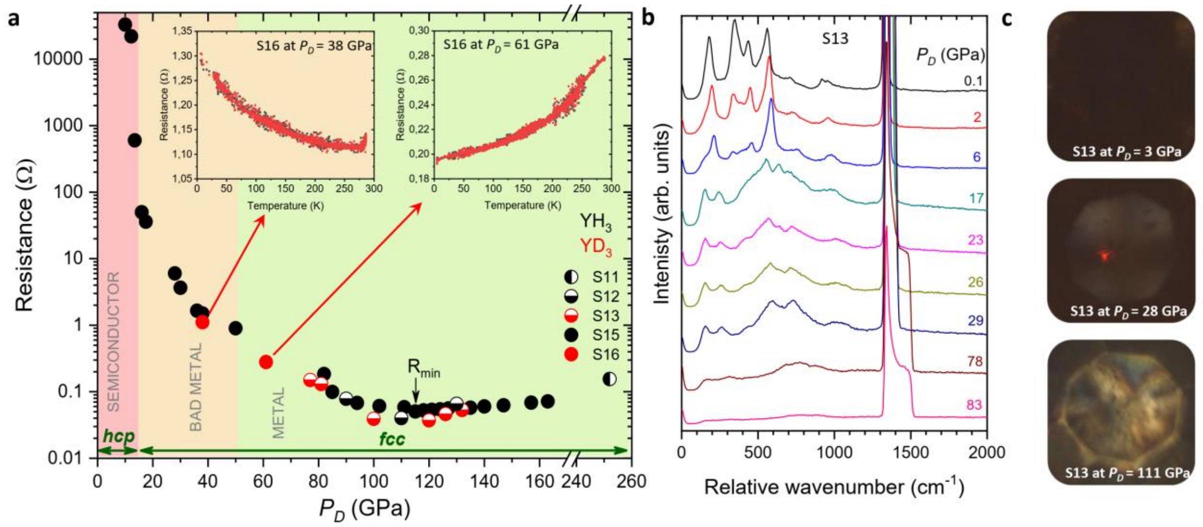

Supplementary Figure 8 Electrical resistance and Raman spectra of $YH_3$and YD_3 at high pressure.
a Resistance dependence on pressure for YH_3 (black symbols) and YD_3 (red symbols) at 295 K. The insets show the typical temperature dependence of the resistance after metallization at high pressure (sample 16 at P_D=38 and 61 GPa). **b** The disappearance of the Raman spectrum of YD_3 in sample 13 during metallization on compression. **c** Photos of sample 13 at several pressures demonstrating the appearance of a metallic lustre.

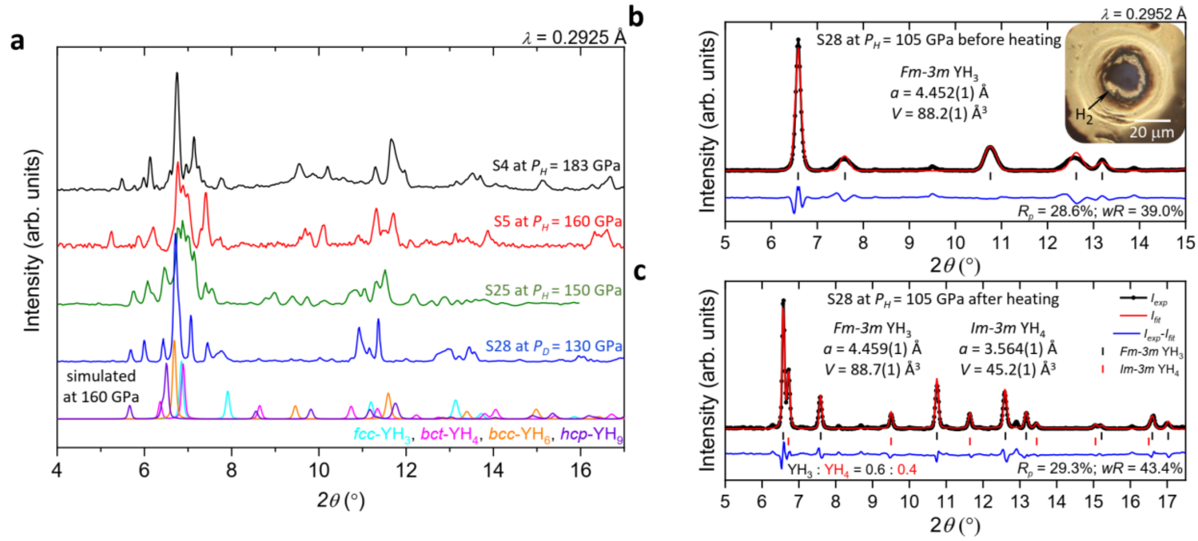

Supplementary Figure 9 Crystalline phases formed in the yttrium-hydrogen system under deficiency of H_2, after poor laser heating or at pressures below 150 GPa.
a Several X-ray powder diffraction patterns of unidentified crystalline phases collected either from poorly heated areas near the sputtered leads (samples 4 and 5), from samples with evident deficiency of H_2 (sample 25), or at pressure below 150 GPa (sample 28). The simulated patterns for the *Fm-3m* YH_3, *I4/mmm* YH_4, *Im-3m* YH_6 and *P6₃/mmc* YH_9 phases are given at the bottom for comparison. **b** Formation of the *Fm-3m* YH_3 phase in sample 28 after exposing Y to H_2 at P_H=105 GPa at ambient temperature for three days. **c** Formation of the new *Im-3m* phase with a tentative composition of YH_4 in sample 28 after subsequent pulsed laser heating at 1500(50) K. Black circles, red and blue curves correspond to the experimental data, Rietveld fits and residues, respectively. Black and red ticks show the calculated peak positions for the *Fm-3m* YH_3 and *Im-3m* YH_4 phases, respectively.

References

1. Eremets, M. I. & Troyan, I. A. Conductive dense hydrogen. *Nat Mater* **10**, 927 (2011).
2. Eremets, M. I. Megabar high-pressure cells for Raman measurements. *J. Raman Spectrosc.* **34**, 515 (2003).
3. Dorfman, S. M., Prakapenka, V. B., Meng, Y. & Duffy, T. S. Intercomparison of pressure standards (Au, Pt, Mo, MgO, NaCl and Ne) to 2.5 Mbar. *J. Geophys. Res.* **117**, B08210 (2012).
4. Vinet, P., Ferrante, J., Rose, J. H. & Smith, J. R. Compressibility of solids. *J. Geophys. Res.* **92**, 9319-9325 (1987).
5. Troyan, I. A. et al. Anomalous high-temperature superconductivity in YH_6. *Adv. Mater.* **33**, 2006832 (2021).
6. Snider, E. et al. Synthesis of Yttrium Superhydride Superconductor with a Transition Temperature up to 262 K by Catalytic Hydrogenation at High Pressures. *Phys. Rev. Lett.* **126**, 117003 (2021).
